# Development and Validation of Epigenetic Modification-Related Signals for the Diagnosis and Prognosis of Hepatocellular Carcinoma

**DOI:** 10.3389/fonc.2021.649093

**Published:** 2021-06-17

**Authors:** Maoqing Lu, Sheng Qiu, Xianyao Jiang, Diguang Wen, Ronggui Zhang, Zuojin Liu

**Affiliations:** ^1^ Department of Endocrinology, The Second Affiliated Hospital of Chongqing Medical University, Chongqing, China; ^2^ Department of Urology, The Second Affiliated Hospital of Chongqing Medical University, Chongqing, China; ^3^ Department of Otorhinolaryngology, The First Affiliated Hospital of Chongqing Medical University, Chongqing, China; ^4^ Department of Hepatobiliary Surgery, The Second Affiliated Hospital of Chongqing Medical University, Chongqing, China

**Keywords:** bioinformatics, hepatocellular carcinoma, epigenetic modification, prognosis, diagnosis, candidate drugs

## Abstract

**Background:**

Increasing evidence has indicated that abnormal epigenetic factors such as RNA m6A modification, histone modification, DNA methylation, RNA binding proteins and transcription factors are correlated with hepatocarcinogenesis. However, it is unknown how epigenetic modification-associated genes contribute to the occurrence and clinical outcome of hepatocellular carcinoma (HCC). Thus, we constructed the epigenetic modification-associated models that may enhance the diagnosis and prognosis of HCC.

**Methods:**

In this study, we focused on the clinical value of epigenetic modification-associated genes for HCC. Our gene expression data were collected from TCGA and HCC data sets from the GEO database to ensure the reliability of the data. Their functions were analyzed by bioinformatics methods. We used lasso regression, Support vector machine (SVM), logistic regression and Cox regression to construct the diagnostic and prognostic models. We also constructed a nomogram of the practicability of the above-mentioned prognostic model. The above results were verified in an independent liver cancer data set from the ICGC database and clinical samples. Furthermore, we carried out pan-cancer analysis to verify the specificity of the above model and screened a wide range of drug candidates.

**Results:**

Many epigenetic modification-associated genes were significantly different in HCC and normal liver tissues. The gene signatures showed a good ability to predict the occurrence and survival of HCC patients, as verified by DCA and ROC curve analysis.

**Conclusion:**

Gene signatures based on epigenetic modification-associated genes can be used to identify the occurrence and prognosis of liver cancer.

## Introduction

Hepatocellular carcinoma (HCC) is one of the most common and fatal malignancies in the world, and the number of HCC cases increases gradually by approximately 4% every year (1, 2). Although early HCC can be treated by tumor resection and liver transplantation, the median survival time of this disease is only a few months (3, 4). The poor prognosis and short lifetime of HCC, to some extent, are dependent on late diagnosis and lack of an effective treatment plan because early HCC has no specific symptoms.

The diagnosis of HCC mainly depends on biopsy and imaging evidence (5, 6). However, due to technical difficulties, the above diagnostic indicators may be affected by subjective factors, resulting in false positive or false negative rates (7). The development of new diagnostic technology can better assist traditional diagnostic methods and help to improve the detection rate of early liver cancer patients. Accurate judgment of patient prognosis is helpful for guiding clinical decision-making and the implementation of precision and personalized medicine. Currently, the prognosis is mainly judged by BCLC and TNM stages, which is insufficient to predict the outcome of patients (8). Therefore, it is necessary to identify effective diagnostic and prognostic biomarkers to help optimize the treatment system of HCC (9). In the past few decades, researchers have identified an increasing number of tumorigenesis mechanisms. One of the breakthroughs is the participation of epigenetic processes in the development of cancer (10).

Epigenetics is a dynamic and heritable modification of independent DNA sequences (11). Abnormal epigenetic changes can destroy the expression balance of oncogenes and tumor suppressor genes and promote tumorigenesis. Common epigenetic modifications include DNA methylation, RNAm6a methylation, and histone acetylation, which are considered the main mechanisms of regulation during cancer progression. Previous studies have focused on the functional exploration of single epigenetic-related genes but lack extensive exploration. Moreover, the value of these genes in the diagnosis and prognosis of liver cancer is still unclear. In this study, we collected five kinds of epigenetically related genes (ERGs), a total of 2397 genes, including RNAm6a modification-related genes, histone modification-related genes, DNA methylation modification-related genes, RNA binding proteins and transcription factors (12–17). To explore them in HCC, we analyzed the differentially expressed epigenetic-related genes in HCC by WGCNA and constructed an apparent regulatory network. To optimize the treatment system of HCC, we integrated epigenetic-related genes to build a diagnostic and prognostic model and compared the signal differences between the high-risk group and the low-risk group by GSEA and GSVA.

## Materials and Methods

### Data Acquisition and Processing

The mRNA expression and patient clinical data were downloaded from the GEO database (GSE14520), TCGA database (TCGA-LIHC data set), GTEx database (GTEX-liver data set) and ICGC database (ICGC-JP data set) (18–21). Gene difference analysis was performed in the form of count data which correction with Limma package. The downstream function analysis is in the form of TPM data. ERGs consisting of m6A-related genes, histone modification-related genes, RNA binding proteins, transcription factors and DNA methylases were collected based on previous literature and databases (**Table S1**) (12–17). Differential expression analysis was performed using “limma” R package. Cytoscape software was used to construct the regulatory network of ERGs and target genes with a criterion of a correlation coefficient greater than 0.75.

### Functional Analysis Based on the WGCNA

The “WGCNA” R package was used for weighted correlation network analysis (WGCNA), and an appropriate soft threshold was chosen to cluster genes with similar coexpression in the same module (22). Clinical data were combined with the above modules to identify clinically meaningful gene clusters. We conducted GO and KEGG analyses with the criteria of a P value < 0.05 and a q value < 0.05 using the R package “enrichplot.”

### Construction of the Diagnostic and Prognostic Model

SVM analysis depended on the “e1071” R package, and lasso regression was conducted to screen diagnostic markers. Diagnostic models were built by logistic regression. Univariate Cox regression analysis was conducted to identify survival-related genes with the criterion of P<0.001. Lasso-multivariate Cox regression analysis was used to establish a prognostic model which performed by “survival” and “glmnet” R package. Kaplan-Meier survival curves were constructed to compare survival-time differences between the high-risk and low-risk groups. We performed the receiver operating characteristic curve (ROC) and decision curve analysis (DCA) to show the accuracy of the model which performed by “survival” R package (23, 24). The model stability was shown using calibration curves.

### Functional Prediction Analysis of the High-PERs and Low-PERs Groups

Based on the median prognostic epigenetic risk score (PERs) in the above TCGA-LIHC data set, the samples were divided into a high-PERs group and a low-PERs group. GSEA_4.0.1 software was used to explore the biological function of the PERs for the two groups based on the hallmark gene set. Gene set variation analysis (GSVA) used the “GSVA” R package and the hallmark gene set to intersect the results.

### Independent Risk Factor Analysis

Univariate and multivariate Cox regression analyses were performed to identify independent risk factors from the above PERs and other clinicopathological factors, such as TNM stage, age, and sex which performed by “survival” R package.

### Evaluation of Drug Effects

The immune response for each sample depends on the tumor immune dysfunction and exclusion (TIDE) database (25). The CTRP2.0 database, which contains the sensitivity data for 481 compounds over 835 CCLs, and the PRISM database, which contains the sensitivity data for 1448 compounds over 482 CCLs, were used to evaluate the efficacy of compounds according to K-nearest neighbor (KNN) imputation. The top and bottom 10% of the high and low subgroups were used as cutting points. Specific methods were described by Shixue Dai et al. (26).

### Western Blots and RT-qPCR

Human HCC samples were obtained *via* surgical resection of HCC patients from The Second Affiliated Hospital of Chongqing Medical University. The protocols were reviewed and supported by the Ethics Committee of The Second Affiliated Hospital of Chongqing Medical University Approval Number (2020): Institutional Review Board (IRB) (STUDY) No. 88, Chongqing, China. Tissue lysis was performed using RIPA lysis buffer (Beyotime Biotechnology, Shanghai, China). Protein was extracted from tissues by centrifugation at 12,000*g* and detected by BCA assays at 562 nm. A rapid gel preparation kit (Beyotime Biotechnology, Shanghai, China) was used to configure the gel distribution at a 140-V constant voltage and 230 mA constant current electroconversion. Primary antibody was incubated at 4°C overnight. Secondary antibody was incubated at 37°C for 1 h. Phosphate-buffered saline was used to wash test strips three times for 10 min each. High-sensitivity ECL (Bio-Rad, USA) solution was used for the exposure strip. ImageJ software was used to analyze the results. RNA extraction and qPCR were performed using RNAiso reagent and a Prime Script™ Reverse Transcriptase kit (TaKaRa, Shiga, Japan) following the manufacturer’s instructions. The specific antibodies and primers are detailed in the supplementary materials.

### Immunohistochemistry

Immunohistochemistry was performed using an immunohistochemistry kit (Solarbio, Beijing, China). Samples were prepared through gradient dehydration of paraffin sections and EDTA antigen thermal repair. Primary antibody was incubated overnight. After three times washes with PBS, secondary antibody was incubated at room temperature for 1 h. Samples were then subjected to DAB staining.

### Statistical Analysis

SPSS and R software were used for statistical analysis. Unless otherwise indicated, a P value < 0.05 was considered statistically significant. Two-tailed Student’s *t* tests were used to test the differences between different groups.

## Results

### Identification of Hub ERGs in HCC

**Figure 1:**
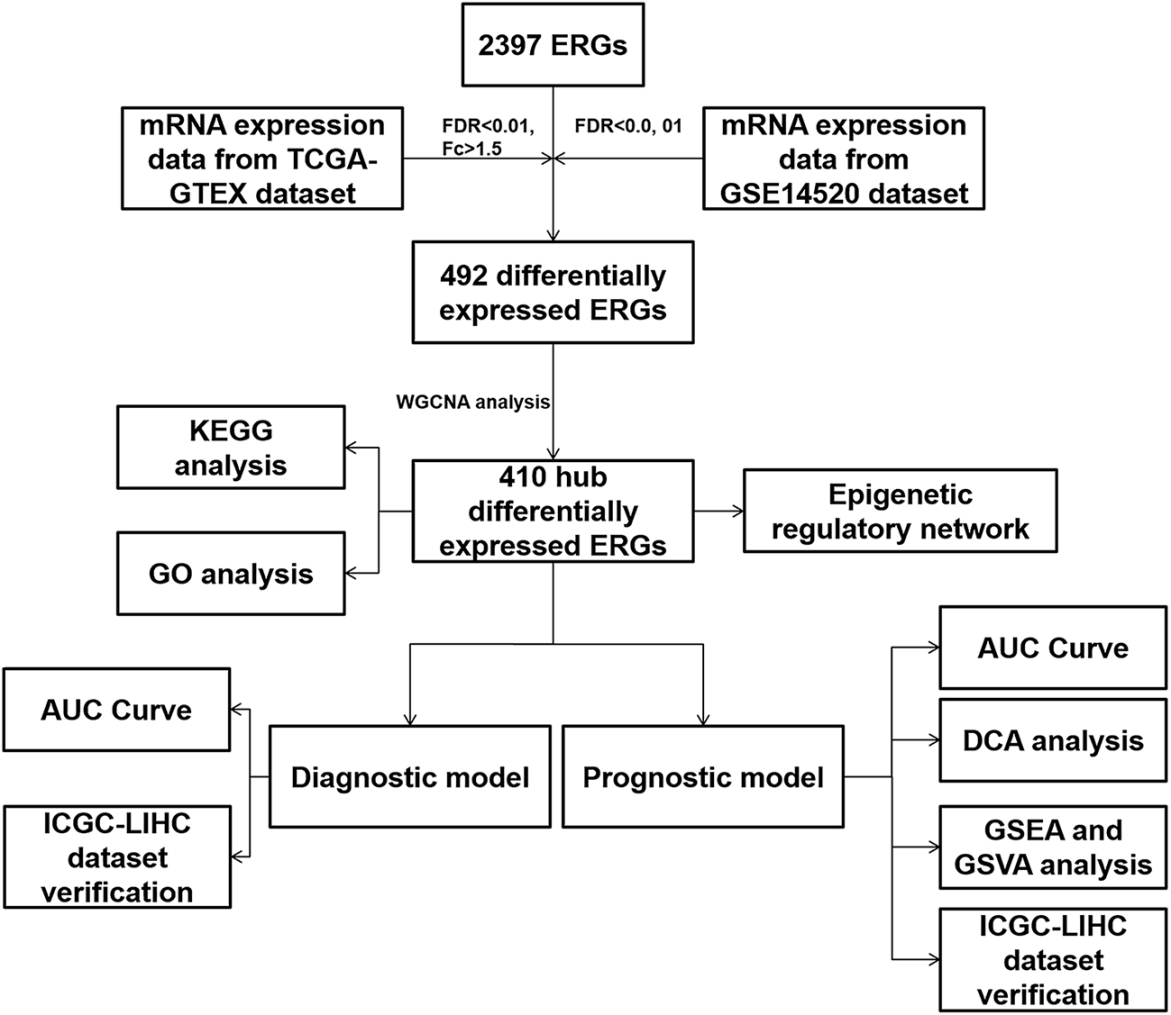
Analysis flowchart of the study.

**Figure 2:**
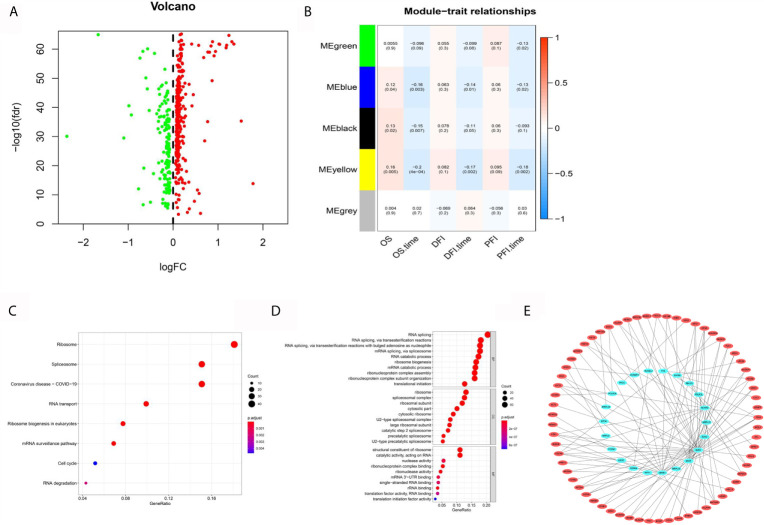
Systematic analysis of epigenetic-related genes. **(A)** Volcano maps for 492 differentially expressed ERGs based on the GTEX-TCGA data set. **(B)** WGCNA analyzed the 492 ERGs combined with TCGA clinical information. **(C)** KEGG analysis of 410 hub ERGs. **(D)** GO analysis of 410 hub ERGs. **(E)** The apparent regulatory network based on a correlation coefficient greater than 0.7.

The analysis flowchart is shown in **Figure 1**. We integrated the 2397 ERGs mentioned above. First, based on the combination of TCGA-LIHC and the GTEX liver sequence data set, we used differential gene analysis because the TCGA-LIHC data set has a few samples of normal liver tissue. The cutoff criterion was FC > 1.5 and FDR < 0.01. Considering the advantages and disadvantages of sequencing and microarrays in evaluating gene expression, we further verified the above results in the largest and most comprehensive online HCC microarray data set, GSE14520, with a cutoff value of FDR < 0.01, which identified 492 differentially expressed ERGs (**Figure 2A**). To further obtain clinically significant ERGs in HCC, we applied WGCNA to analyze the above genes and obtained five modules in HCC; among them, the blue, black, green, and yellow modules were closely related to patient DFI, PFI and OS based on the widely validated TCGA clinical data, resulting in 410 hub ERGs (**Figure 2B**).

### KEGG and GO Analysis and Construction of the Epigenetic Factor Regulatory Network

Kyoto Encyclopedia of Genes and Genomes (KEGG) and Gene Ontology (GO) analyses, which can identify gene functions in a high throughput manner based on previous gene annotation, were performed to explore the functions of 410 hub ERGs. The KEGG pathways included ribosome, spliceosome and RNA transport (**Figure 2C**). In addition, the GO analyses showed that biological process (BP) terms were mainly regulation of RNA splicing, ribonucleoprotein biogenesis, and RNA splicing; the cell component (CC) terms were ribosomal subunit, ribosome and spliceosomal complex; and the molecular function terms showed significant enrichment in structural constituent of ribosome, catalytic activity, acting on RNA and nuclease activity (**Figure 2D**). Furthermore, to determine the regulatory targets of ERGs, we identified the differentially expressed genes in HCC according to the previous analysis and constructed an apparent differentially expressed regulatory network according to a correlation coefficient greater than 0.75 (**Figure 2E**).

### ERGs for the Diagnosis of HCC

**Figure 3:**
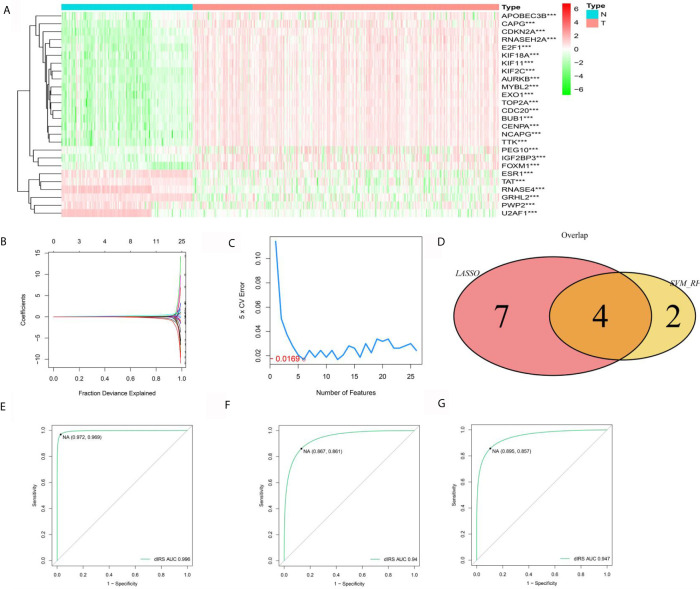
Establishment and validation of the diagnostic signal for HCC. **(A)** Twelve ERGs with significantly different expression based on the GTEX-TCGA data set. **(B)** Lasso regression analysis of the above ERGs. **(C)** Support vector machine analysis of the above ERGs. **(D)** The characteristic value is based on the intersection of SVM and lasso analysis. **(E)** The ROC curve of the diagnostic signal showing the sensitivity and specificity based on the GTEX-TCGA data set. **(F)** The ROC curve of the diagnostic signal showing the sensitivity and specificity based on the ICGC-LIHC data set. **(G)** The ROC curve of the diagnostic signal showing the sensitivity and specificity based on the GSE14520 data set. ***<0.001.

To evaluate the diagnostic value of hub ERGs, we strictly filtered the range of differences, and the cutoff log2FC value was greater than 2.5 based on the TCGA-GTEX data set, which identified 26 ERGs (**Figure 3A**). For the accuracy and refinement of the model, we performed support vector machine (SVM) and least absolute shrinkage and selection operator (lasso) analyses, and four genes were selected from the intersection to construct a diagnostic epigenetic risk score (dERS) model using multivariate logistic regression analysis. The model formula is = (1.245*TOP2A)+ (−1.144*GRHL2)+(1.667*-RNASE4)+(1.963*CDKN2A) (**Figures 3B–D**). In the TCGA-GTEX data set, the receiver operating characteristic curve (ROC) showed an excellent AUC value (0.996), specificity (0.972) and sensitivity (0.969) which cutoff value was −2.51 (**Figure 3E**). We also verified the above diagnostic model with the independent data set ICGC-JP (**Figure 3F**). In addition, we also verified the above diagnostic model in the microarray data set (GSE14520), indicating that the above model is not only suitable for sequencing data, but also suitable for microarray data (**Figure 3G**).

### Experimental Verification of Diagnosis-Associated Genes

**Figure 4:**
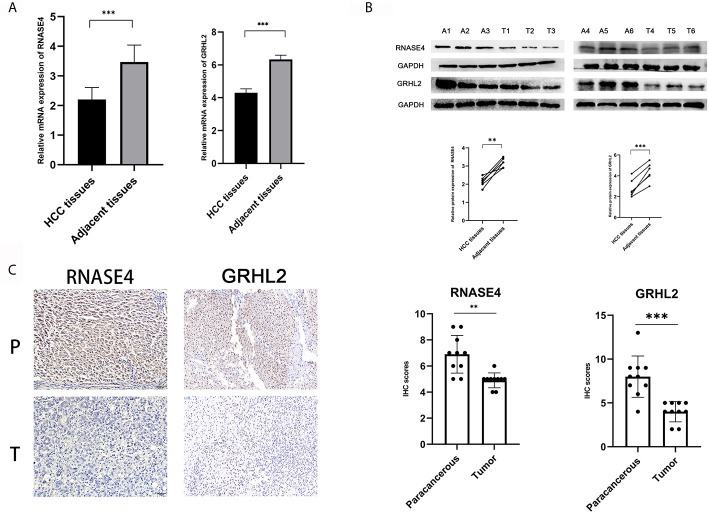
Verification of the expression of diagnosis-related genes. **(A)** RT-qPCR assay for RNASE4 and GRHL2 in HCC (n = 30). **(B)** Western blot assay for RNASE4 and GRHL2 in HCC (n = 6). **(C)** Immunohistochemistry assay for RNASE4 and GRHL2 in HCC (n = 10). **<0.01 ***<0.001.

For the reliability of the data, we further explored the expression of genes related to diagnosis of HCC. Two genes (TOP2A and CDKN2A) were reported by previous studies in HCC (27, 28). Furthermore, we performed RT-qPCR assays, which confirmed the expression of RNASE4 and GRHL2 in 30 pairs of liver cancer and adjacent tissues (**Figure 4A**). At the protein level, Western blot experiments were conducted in which RNASE4 and GRHL2 expression was significantly downregulated in HCC (**Figure 4B**). Immunohistochemistry was also performed in 10 paired of liver cancer and adjacent tissues to furthermore measure the expression of RNASE4 and GRHL2 (**Figure 4C**).

### ERGs for the Prognosis of HCC

**Figure 5:**
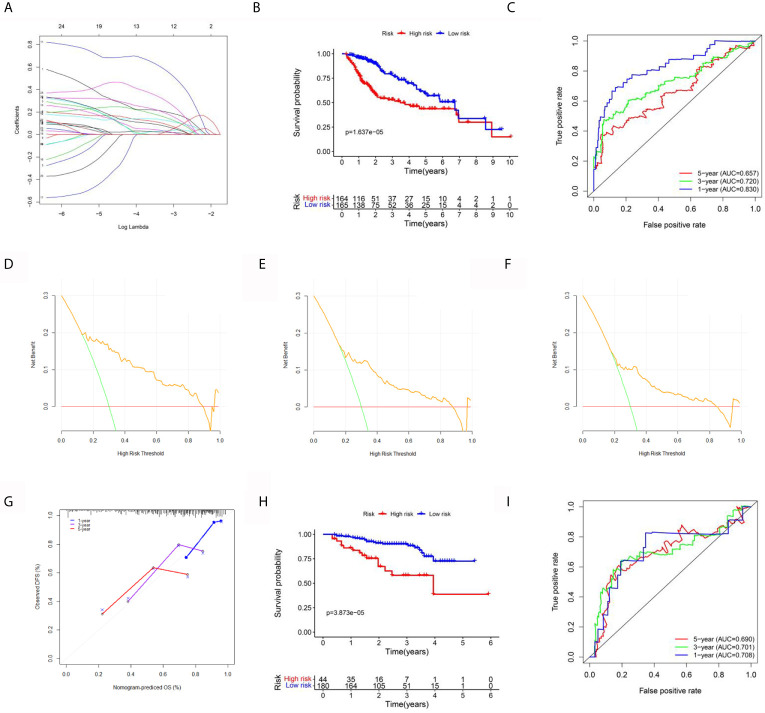
Establishment and validation of the prognostic signal for HCC. **(A)** Lasso regression analysis to identify the characteristic value of the constructed diagnostic signal. **(B)** Survival analysis between the high-risk and low-risk groups. **(C)** The area under the ROC curve (AUC) of PIRs for 1-, 3-, and 5-year OS in the TCGA data set. **(D–F)** The DCA curve of PERs for 1, 3, and 5 years in the TCGA data set. **(G)** The calibration curve of PERs for 1, 3, and 5 years in the TCGA data set. **(H)** Survival analysis between the high-risk and low-risk groups in the ICGC-LIHC data set. **(I)** The AUC of PIRs for 1-, 3-, and 5-year OS in the TCGA data set.

To explore the value of the above hub ERGs in HCC, we conducted univariate Cox regression analysis to identify 27 survival-related genes (P<0.001). Next, we established a prognostic epigenetic risk score (PER) consisting of seven genes by lasso-Cox regression analysis (**Figure 5A**). PERs were calculated for patients in the TCGA-LIHC data set and divided into high-PER and low-PER groups according to the 50% cutoff point. The high-PERs group had shorter survival than the low-PERs group which cutoff value was 10.52 (**Figure 5B**). The AUC values for 1-, 3- and 5-year OS were 0.830, 0.720, and 0.657, respectively (**Figure 5C**). In addition, we performed decision curve analysis (DCA), which showed that PERs provided good benefits to patients (**Figures 5D–F**). The calibration curve also showed excellent stability (**Figure 5G**). We also compared the differences of clinical features between the high and low PERs groups which verified the reliability of the results from the inside of data set (**Table 1**). The above survival-related model was also verified with an independent data set, the ICGC-JP data set, and the result was consistent with the TCGA-LIHC data set which cutoff value was 10.52 (**Figures 5H, I** **)**.

**Table 1:** Clinical characteristics between the high- and low-risk groups.

	TCGA-LIHC		
**Characters**	**Low risk**	**High risk**	**P value**
Gender			.749
Female	53	50	
Male	112	114	
Age(years)			.164
≤55	51	63	
>55	113	101	
Grade			<.001
G1+G2	118	87	
G3+G4	44	75	
TNM Stage			.003
I-II	126	103	
III-IV	28	51	
T Stage			.008
T1+T2	132	111	
T3+T4	31	52	
N Stage			.081
N0	116	113	
N1	0	3	
M Stage			.081
M0	117	120	
M1	3	0	

### Analysis of the Clinical Characteristics for the PERs Groups

**Figure 6:**
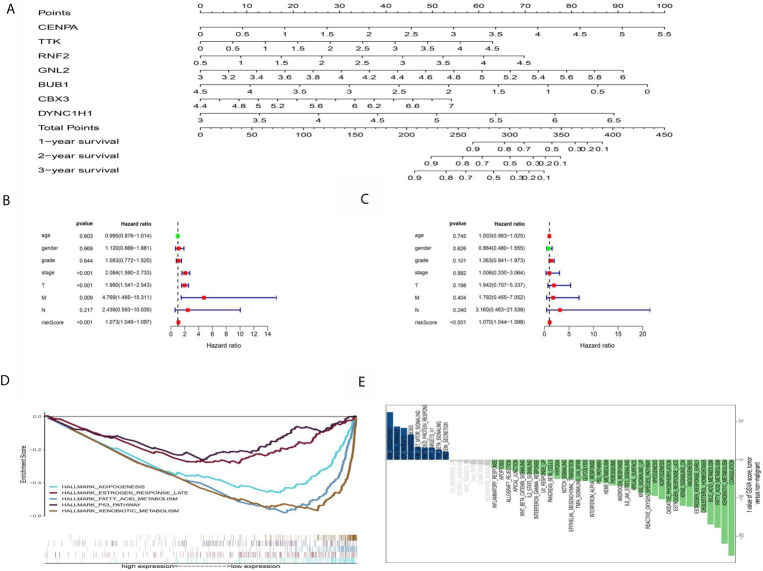
Functional analysis between the high-PERs and low-PERs groups. **(A)** The nomograms for predicting the 1-, 3-, and 5-year survival rates in the TCGA data set. **(B)** Univariate Cox regression analysis for PERs in the TCGA data set. **(C)** Multivariate Cox regression analysis for PERs in the TCGA data set. **(D)** GSEA between the high-PERs and low-PERs groups in the TCGA data set. **(E)** GSVA between the high-PERs and low-PERs groups in the TCGA data set.

For clinical practicability, a nomogram was drawn to predict 1-year, 3-year, and 5-year survival (**Figure 6A**). Through univariate and multivariate regression analyses, we identified PERs as an independent prognostic factor for HCC (**Figures 6B, C** **)**. The differences in clinical characteristics between the high-PERs and low-PERs groups were analyzed in the above data sets, and the accuracy of the model was further verified based on the data set. Based on the TCGA-LIHC data set, the differentially expressed signaling pathways between the high-PERs and low-PERs groups were identified by using the GSEA and GSVA algorithms to obtain the intersection (**Figures 6D, E**, and **S1**) (29, 30), which helps elucidate the difference between the high-PERs and low-PERs groups.

### Pan-Cancer Analysis

**Figure 7:**
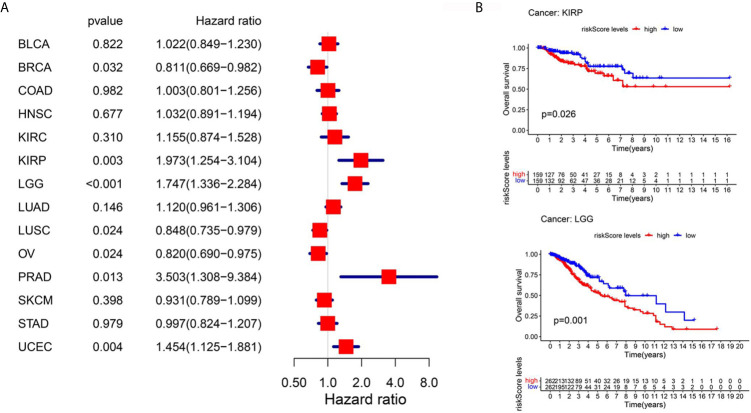
Pan-cancer analysis using TCGA database. **(A)** Univariate Cox regression analysis for 14 kinds of tumors. **(B)** Km survival curve analysis between the high-PERs and low-PERs groups, with a P value <0.05.

One of the advantages of bioinformatics analysis is that it can obtain a variety of tumor data for analysis. To explore the applicability of these prognostic models in other tumors, we collected 14 tumor (BLCA, BRCA, COAD, HNSC, KIRC, KIRP, LGG, LUAD, LUSC, OV, PRAD, SKCM, STAD, UCEC) data sets with sample sizes greater than 300 in TCGA database. First, we performed univariate Cox regression analysis which only a few tumors, such as KIRP, and OV were suitable for the above survival model (**Figure 7A**). However, KM survival analysis showed that only LGG and KIRP had significant differences (**Figure 7B**), which may be attributed to different statistical methods, but both of them indicated the liver cancer specificity of the above models

### Bioinformatics Prediction of Drug Candidates

**Figure 8:**
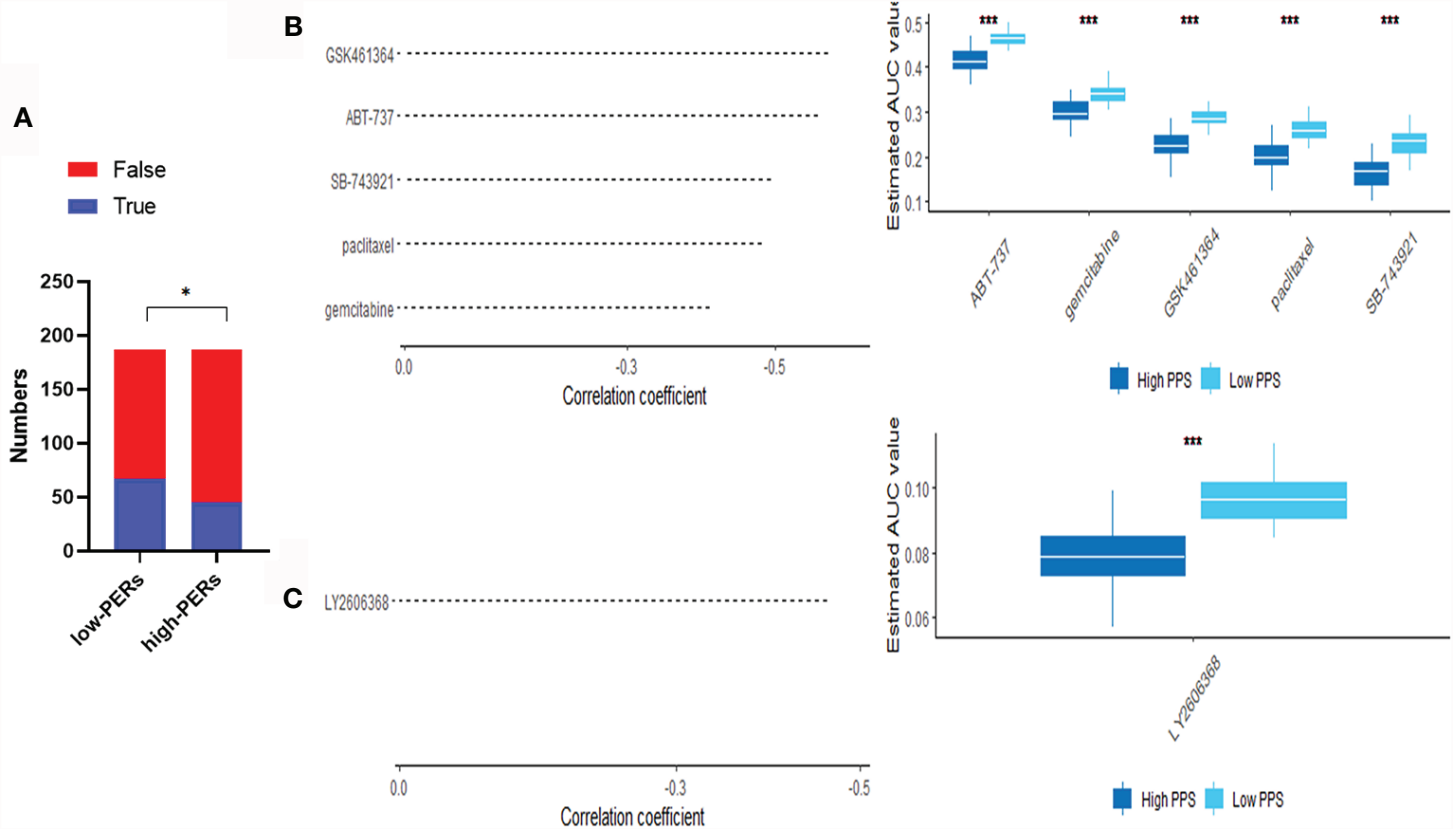
Evaluation of drug effects. **(A)** The evaluation of immunotherapy based on the TIDE database. **(B)** The results of Spearman’s correlation analysis and differential drug response analysis of three CTRP-derived compounds. **(C)** The results of Spearman’s correlation analysis and differential drug response analysis of one PRISM-derived compounds. Note that lower values on the y-axis of boxplots represented greater drug sensitivity. *<0.05, ***<0.001.

To further evaluate the effects of various drugs on the different PERs subgroups, we evaluated each patient’s response to immunotherapy using the TIDE database. We found that the low-PERs group was more suitable for immunotherapy (**Figure 8A**). To identify candidate drugs for the high-PERs group, we comprehensively analyzed the PRISM and CTRP2.0 databases and found that four compounds may be effective in the low-PERs group. The resulted based on the PRISM found that five drugs (ABT-737, gemcitabine, GSK461364, paclitaxel and SB-743921) was better response in low-PERs group (**Figure 8B** **)** and the resulted based on the CTRP2.0 found that one drug (LY2606368) was better response in low-PERs group (**Figure 8C**). These results can provide some guidance for clinical treatment.

## Discussion

Although the level of medical diagnosis and treatment has been improved in recent years, the accuracy of diagnosis and the survival rate and prognosis of HCC are still poor (31). Future research should identify clinically significant genes, predict their functions and explore their prognostic value based on bioinformatics. At present, there is a lack of effective biomarkers with high accuracy for the diagnosis and prognosis of HCC (32). However, in the past, bioinformatics research often focused on a single database or only focused on prognostic value, which had some limitations. In recent decades, scientific workers have identified many aspects of epigenetic modification regulating gene expression that interfere with tumor progression. DNA methylation, RNA m6A methylation, histone-related modification and so on are hot spots of tumor research. Previous studies have mainly focused on the impact of single epigenetic-related genes on tumor prognosis and function. Based on bioinformatics analysis, the above genes can be widely recognized for the diagnosis and prognosis of liver cancer, and functional prediction can be carried out, which can provide help for later experimental research. In addition, the regulation of gene expression by epigenetic regulators is closely related to transcription factors and RNA binding proteins; thus, we collected five kinds of ERGs: RNA m6A modification-related genes, histone modification-related genes, DNA methylation-related genes, RNA binding proteins and transcription factors. Based on these genes, we successfully constructed a stable and reliable diagnostic and prognostic model for HCC, which was verified by an independent data set.

In this study, to ensure the accuracy of the analysis, we identified 492 differentially expressed ERGs according to the combination analysis of three online data sets. WGCNA can cluster disease genes according to the correlation of their intrinsic expression and identify key gene modules by combining them with clinical phenotypes (22). TCGA database is the most authoritative tumor database, including a variety of histological data and clinical information. OS, DFI, and PFI are recommended for survival-related analysis of the TCGA-LIHC data set. Thus, using WGCNA, a total of five coexpression modules were separated, and we chose the blue, black, green and yellow modules for the next study because they are closely related to survival and acquired 410 hub IRGs. KEGG and GO analysis can fully elucidate gene set functions compared with individual studies on gene function (33, 34). We performed KEGG and GO analyses, and the results showed that the above genes were mainly enriched in RNA splicing and ribonucleoprotein complex biogenesis (35, 36). Previous studies have shown that these physiological activities are closely related to tumorigenesis. Furthermore, we constructed a regulatory network of ERGs and target genes, which can preliminarily and comprehensively describe the regulatory relationship of these genes.

Next, we investigated the diagnostic and prognostic value of the above-mentioned hub ERGs. Support vector machines can not only be used to build classification models but can also be used to screen important variables to build a simpler and more accurate model (37). Based on the TCGA-GTEX data set, we found important variables through SVM and lasso analysis and established a diagnostic model consisting of four ERGs for HCC by logistic regression with a specificity of 0.972 and sensitivity of 0.969. We also validated the above diagnostic model in an independent data set, the ICGC-LIHC data set, which had a specificity of 0.867 and a sensitivity of 0.861. There were a few differences, which may be attributed to the difference in the source of the included groups. The results showed the reliability and stability of the diagnostic model.

Furthermore, we screened seven genes (CENPA, TTK, RNF2, GNL2, BUB1, CBX3, and DYNC1H1) that were significantly associated with prognosis of HCC. As a transcription factor, CENPA is up-regulated in a variety of tumors. It is considered to be one of the potential therapeutic targets for liver cancer, and is associated with HBV infection (38). TTK is an RNA-binding protein which closely related to the proliferation and metastasis of HCC (39). RNF2 is a transcription factor, which has been reported to be related to the tumor progression (40). GNL2, an RNA-binding protein, is lack of reports in tumor and has been reported to be involved in the biological development (41). At present, BUB1 is mostly studied in liver cancer, involving multiple signal transduction pathways (42). Although the mechanism remains unclear, CBX3 is reported to be involved in the development of HCC (43). DYNC1H1 is reported to be involved in ERK related signaling pathway (44). Based on this, we established a prognostic risk model including ten ERGs by lasso-Cox analysis. The AUC values for 1-, 3-, and 5-year OS were 0.830, 0.720, and 0.657 in the TCGA-LIHC data set and 0.708, 0.701, and 0.690 in the ICGC-JP data set, respectively. These results support the reliability of the prognostic model. For the reliability of functional prediction, we also used two methods (GSEA and GSVA) to identify different signaling pathways between the high-risk group and the low-risk group. The results showed that hypoxia, glycolysis, apoptosis, and other signaling pathways were enriched in the low-risk group and may be potential therapeutic targets.

There are significant metabolic abnormalities of fatty acids in tumors. The significant increase in lipid droplets in tumor cells can promote the transformation of tumor mesenchymal cells. Reducing the accumulation of fatty acids in cells can reduce the migration and invasion of tumor cells (45). Hypoxia also participates in the formation of a protumor microenvironment (46). Glycolysis is regarded as one of the key metabolic characteristics of malignant tumor (47). Apoptosis is one of the main means of anti-tumor (48). These studies suggest that the differential expression of multiple signaling pathways in the high-PERs and low-PERs groups may be used for potential therapeutic strategies, which also confirmed the reliability of our prognostic model within the gene set. In addition, we used the TIDE database algorithm to evaluate our data and found that immunotherapy may be more reliable for low-PERs groups. The KNN algorithm combined with a pharmacodynamic database was used to screen the effective compounds for the low-PERs group on a large scale, which can promote the development of clinical treatment in the future (49).

## Conclusion

We first comprehensively analyzed ERGs in liver cancer. Compared with previous bioinformatics studies that only constructed a prognostic model, we discuss the influence of gene sets on diagnosis and prognosis. These results can help elucidate HCC development and can contribute to future experimental research. More importantly, we constructed two novel biomarkers to guide the management of liver cancer. Although the major limitation of this study is the lack of verification by enough experimental data, we tried our best to ensure the reliability of the data through multiple data sets or multiple algorithms and conducted appropriate clinical sample validation. Overall, this study will be valuable for the treatment and diagnosis of HCC.

## Data Availability Statement

The original contributions presented in the study are included in the article/**Supplementary Material**. Further inquiries can be directed to the corresponding author.

## Ethics Statement

The studies involving human participants were reviewed and approved by the ethics committee of The Second Affiliated Hospital of Chongqing Medical University. The patients/participants provided their written informed consent to participate in this study.

## Author Contributions

MQL, DGW, SQ, XYJ and ZJL performed the research and wrote the draft. MQL and XYJ is responsible for the following manuscript modification. DW and SQ collected and analyzed the data. ZJL is the guarantor. All authors contributed to the article and approved the submitted version. The contributions of MQL, SQ and XYJ were the same, and the order of authors was random.

## Funding

This project was supported the National Science Foundation of China (no. 81170442, 81470899, 81702357, 8207034238) and Chongqing Natural Science Foundation (cstc2020jcyj-msxm0631, cstc2019jcyj-zdxmX0027).

## Conflict of Interest

The authors declare that the research was conducted in the absence of any commercial or financial relationships that could be construed as a potential conflict of interest.

## Abbreviations

HCC, Hepatocellular carcinoma; TCGA, The Cancer Genome Atlas; GEO, Gene Expression Omnibus; KEGG, Kyoto Encyclopedia of Genes and Genomes; GSEA, Gene Set Enrichment Analyses; GSVA, Gene Set Variation Analysis; ICGC, International Cancer Genome Consortium.

## References

[1] Tang A Hallouch O Chernyak V Kamaya A Sirlin CB. Epidemiology of Hepatocellular Carcinoma: Target Population for Surveillance and Diagnosis. Abdom Radiol (NY) (2018) 43(1):13–25. 10.1007/s00261-017-1209-1 28647765

[2] Liu Y Zhang X Zhang J Tan J Li J Song Z. Development and Validation of a Combined Ferroptosis and Immune Prognostic Classifier for Hepatocellular Carcinoma. Front Cell Dev Biol (2020) 8:596679. 10.3389/fcell.2020.596679 33425905 PMC7785857

[3] Yang JD Hainaut P Gores GJ Amadou A Plymoth A Roberts LR. A Global View of Hepatocellular Carcinoma: Trends, Risk, Prevention and Management. Nat Rev Gastroenterol Hepatol (2019) 16(10):589–604. 10.1038/s41575-019-0186-y 31439937 PMC6813818

[4] Ono A Aikata H Yamauchi M Kodama K Ohishi W Kishi T. Circulating Cytokines and Angiogenic Factors Based Signature Associated With the Relative Dose Intensity During Treatment in Patients With Advanced Hepatocellular Carcinoma Receiving Lenvatinib. Ther Adv Med Oncol (2020) 12:1758835920922051. 10.1177/1758835920922051 32547646 PMC7249573

[5] Russo FP Imondi A Lynch EN Farinati F. When and How Should We Perform a Biopsy for HCC in Patients With Liver Cirrhosis in 2018? A Rev Dig Liver Dis (2018) 50(7):640–6. 10.1016/j.dld.2018.03.014 29636240

[6] Yang Y Liu C Qi L Zhao T Feng Y Ai X. Diagnosis of Pre-HCC Disease by Hepatobiliary-Specific Contrast-Enhanced Magnetic Resonance Imaging: A Review. Dig Dis Sci (2020) 65(9):2492–502. 10.1007/s10620-019-05981-0 31808004

[7] Ayuso C Rimola J Vilana R Burrel M Darnell A García-Criado Á. Diagnosis and Staging of Hepatocellular Carcinoma (HCC): Current Guidelines. Eur J Radiol (2018) 101:72–81. 10.1016/j.ejrad.2018.01.025 29571804

[8] Zhang F Wang K Du P Yang W He Y Li T. Risk of Stroke in Cancer Survivors: A Meta-Analysis of Population-Based Cohort Studies. Neurology (2021) 96(4):e513–26. 10.1212/WNL.0000000000011264 33277416

[9] Sun M Liu X Xia L Chen Y Kuang L Gu X. A Nine-lncRNA Signature Predicts Distant Relapse-Free Survival of HER2-Negative Breast Cancer Patients Receiving Taxane and Anthracycline-Based Neoadjuvant Chemotherapy. Biochem Pharmacol (2020) 189:114285. 10.1016/j.bcp.2020.114285 33069665

[10] Nacev BA Jones KB Intlekofer AM Yu J Allis CD Tap WD. The Epigenomics of Sarcoma. Nat Rev Cancer (2020) 20(10):608–23. 10.1038/s41568-020-0288-4 PMC8380451 32782366

[11] Lu Y Chan YT Tan HY Li S Wang N Feng Y. Epigenetic Regulation in Human Cancer: The Potential Role of Epi-Drug in Cancer Therapy. Mol Cancer (2020) 19(1):79. 10.1186/s12943-020-01197-3 32340605 PMC7184703

[12] Liu T Ortiz JA Taing L Meyer CA Lee B Zhang Y. Cistrome: An Integrative Platform for Transcriptional Regulation Studies. Genome Biol (2011) 12(8):R83. 10.1186/gb-2011-12-8-r83 21859476 PMC3245621

[13] Williams K Christensen J Helin K. DNA Methylation: TET Proteins-Guardians of CpG Islands. EMBO Rep (2011) 13(1):28–35. 10.1038/embor.2011.233 22157888 PMC3246258

[14] Khare SP Habib F Sharma R Gadewal N Gupta S Galande S. Histome–a Relational Knowledgebase of Human Histone Proteins and Histone Modifying Enzymes. Nucleic Acids Res (2012) 40:D337–42. 10.1093/nar/gkr1125 PMC3245077 22140112

[15] Gujar H Weisenberger DJ Liang G. The Roles of Human Dna Methyltransferases and Their Isoforms in Shaping the Epigenome. Genes (Basel) (2019) 10(2):172. 10.3390/genes10020172 PMC6409524 30813436

[16] Chen M Wong CM. The Emerging Roles of N6-Methyladenosine (m6A) Deregulation in Liver Carcinogenesis. Mol Cancer (2020) 19(1):44. 10.1186/s12943-020-01172-y 32111216 PMC7047367

[17] Li K Guo ZW Zhai XM Yang XX Wu YS Liu TC. RBPTD: A Database of Cancer-Related RNA-Binding Proteins in Humans. Database (Oxford) (2020) 2020:baz156. 10.1093/database/baz156 32047888 PMC7012770

[18] Barrett T Edgar R. Reannotation of Array Probes At NCBI’s GEO Database. Nat Methods (2008) 5(2):117. 10.1038/nmeth0208-117b 18235428

[19] GTEx Consortium. The Genotype-Tissue Expression (GTEx) Project. Nat Genet (2013) 45(6):580–5. 10.1038/ng.2653 PMC4010069 23715323

[20] Liu J Lichtenberg T Hoadley KA Poisson LM Lazar AJ Cherniack AD. An Integrated Tcga Pan-Cancer Clinical Data Resource to Drive High-Quality Survival Outcome Analytics. Cell (2018) 173(2):400–16.e11. 10.1016/j.cell.2018.02.052 29625055 PMC6066282

[21] Cortés-Ciriano I Lee JJ Xi R Jain D Jung YL Yang L. Comprehensive Analysis of Chromothripsis in 2,658 Human Cancers Using Whole-Genome Sequencing. Nat Genet (2020) 52(3):331–41. 10.1038/s41588-019-0576-7 PMC7058534 32025003

[22] Zhao W Langfelder P Fuller T Dong J Li A Hovarth S. Weighted Gene Coexpression Network Analysis: State of the Art. J Biopharm Stat (2010) 20(2):281–300. 10.1080/10543400903572753 20309759

[23] Obuchowski NA Bullen JA. Receiver Operating Characteristic (ROC) Curves: Review of Methods With Applications in Diagnostic Medicine. Phys Med Biol (2018) 63(7):07TR01. 10.1088/1361-6560/aab4b1 29512515

[24] Van Calster B Wynants L Verbeek J Verbakel JY Christodoulou E Vickers AJ. Reporting and Interpreting Decision Curve Analysis: A Guide for Investigators. Eur Urol (2018) 74(6):796–804. 10.1016/j.eururo.2018.08.038 30241973 PMC6261531

[25] Fu J Li K Zhang W Wan C Zhang J Jiang P. Large-Scale Public Data Reuse to Model Immunotherapy Response and Resistance. Genome Med (2020) 12(1):21. 10.1186/s13073-020-0721-z 32102694 PMC7045518

[26] Yang C Huang X Li Y Chen J Lv Y Dai S. Prognosis and Personalized Treatment Prediction in TP53-Mutant Hepatocellular Carcinoma: An in Silico Strategy Towards Precision Oncology. Brief Bioinform (2020) 22(3):bbaa164. 10.1093/bib/bbaa164 32789496

[27] Cai H Shao B Zhou Y Chen Z. High Expression of TOP2A in Hepatocellular Carcinoma Is Associated With Disease Progression and Poor Prognosis. Oncol Lett (2020) 20(5):232. 10.3892/ol.2020.12095 32968454 PMC7500035

[28] Hui AM Sakamoto M Kanai Y Ino Y Gotoh M Yokota J. Inactivation of p16INK4 in Hepatocellular Carcinoma. Hepatology (1996) 24:575–9. 10.1002/hep.510240319 8781327

[29] Lappano R Talia M Cirillo F Rigiracciolo DC Scordamaglia D Guzzi R. The IL1β-IL1R Signaling Is Involved in the Stimulatory Effects Triggered by Hypoxia in Breast Cancer Cells and Cancer-Associated Fibroblasts (CAFs). J Exp Clin Cancer Res (2020) 39(1):153. 10.1186/s13046-020-01667-y 32778144 PMC7418191

[30] Wang C Yang Y Yin L Wei N Hong T Sun Z. Novel Potential Biomarkers Associated With Epithelial to Mesenchymal Transition and Bladder Cancer Prognosis Identified by Integrated Bioinformatic Analysis. Front Oncol (2020) 10:931. 10.3389/fonc.2020.00931 32695668 PMC7338771

[31] Huang A Yang XR Chung WY Dennison AR Zhou J. Targeted Therapy for Hepatocellular Carcinoma. Signal Transduct Target Ther (2020) 5(1):146. 10.1038/s41392-020-00264-x 32782275 PMC7419547

[32] Baudi I Inoue T Tanaka Y. Novel Biomarkers of Hepatitis B and Hepatocellular Carcinoma: Clinical Significance of HBcrAg and M2bpgi. Int J Mol Sci (2020) 21(3):349. 10.3390/ijms21030949 PMC7037346 32023902

[33] Chen L Zhang YH Lu G Huang T Cai YD. Analysis of Cancer-Related lncRNAs Using Gene Ontology and KEGG Pathways. Artif Intell Med (2017) 76:27–36. 10.1016/j.artmed.2017.02.001 28363286

[34] Zheng F Zhang W Chu X Dai Y Li J Zhao H. Genome Sequencing of Strain Cellulosimicrobium Sp. TH-20 With Ginseng Biotransformation Ability. 3 Biotech (2017) 7(4):237. 10.1007/s13205-017-0850-2 PMC5505874 28698996

[35] Domínguez-Sánchez MS Sáez C Japón MA Aguilera A Luna R. Differential Expression of THOC1 and ALY mRNP Biogenesis/Export Factors in Human Cancers. BMC Cancer (2011) 11:77. 10.1186/1471-2407-11-77 21329510 PMC3050854

[36] Kawamura N Nimura K Saga K Ishibashi A Kitamura K Nagano H. Sf3b2-Mediated RNA Splicing Drives Human Prostate Cancer Progression. Cancer Res (2019) 79(20):5204–17. 10.1158/0008-5472.CAN-18-3965 31431456

[37] Huang S Cai N Pacheco PP Narrandes S Wang Y Xu W. Applications of Support Vector Machine (SVM) Learning in Cancer Genomics. Cancer Genomics Proteom (2018) 15(1):41–51. 10.21873/cgp.20063 PMC5822181 29275361

[38] Bayo J Fiore EJ Dominguez LM Real A Malvicini M Rizzo M. A Comprehensive Study of Epigenetic Alterations in Hepatocellular Carcinoma Identifies Potential Therapeutic Targets. J Hepatol (2019) 71:78–90. 10.1016/j.jhep.2019.03.007 30880225

[39] Miao R Wu Y Zhang H Zhou H Sun X Csizmadia E. Utility of the Dual-Specificity Protein Kinase TTK as a Therapeutic Target for Intrahepatic Spread of Liver Cancer. Sci Rep (2016) 6:33121. 10.1038/srep33121 27618777 PMC5020615

[40] Qu C Qu Y. Down-Regulation of Salt-Inducible Kinase 1 (SIK1) Is Mediated by RNF2 in Hepatocarcinogenesis. Oncotarget (2017) 8:3144–55. 10.18632/oncotarget.13673 PMC5356871 27911266

[41] Paridaen JT Janson E Utami KH Pereboom TC Essers PB van Rooijen C. The Nucleolar GTP-binding Proteins Gnl2 and Nucleostemin Are Required for Retinal Neurogenesis in Developing Zebrafish. Dev Biol (2011) 355:286–301. 10.1016/j.ydbio.2011.04.028 21565180

[42] Zhang H Chu K Zheng C Ren L Tian R. Pseudogene DUXAP8 Promotes Cell Proliferation and Migration of Hepatocellular Carcinoma by Sponging MiR-490-5p to Induce Bub1 Expression. Front Genet (2020) 11:666. 10.3389/fgene.2020.00666 32849765 PMC7396656

[43] Zhong X Kan A Zhang W Zhou J Zhang H Chen J. Cbx3/Hp1γ Promotes Tumor Proliferation and Predicts Poor Survival in Hepatocellular Carcinoma. Aging (Albany NY) (2019) 11:5483–97. 10.18632/aging.102132 PMC6710055 31375643

[44] Garrett CA Barri M Kuta A Soura V Deng W Fisher EM. DYNC1H1 Mutation Alters Transport Kinetics and ERK1/2-cFos Signalling in a Mouse Model of Distal Spinal Muscular Atrophy. Brain (2014) 137:1883–93. 10.1093/brain/awu097 24755273

[45] Coutzac C Jouniaux JM Paci A Schmidt J Mallardo D Seck A. Systemic Short Chain Fatty Acids Limit Antitumor Effect of CTLA-4 Blockade in Hosts With Cancer. Nat Commun (2020) 11:2168. 10.1038/s41467-020-16079-x 32358520 PMC7195489

[46] Li Y Zhao L Li XF. The Hypoxia-Activated Prodrug TH-302: Exploiting Hypoxia in Cancer Therapy. Front Pharmacol (2021) 12:636892. 10.3389/fphar.2021.636892 33953675 PMC8091515

[47] Ganapathy-Kanniappan S. Molecular Intricacies of Aerobic Glycolysis in Cancer: Current Insights Into the Classic Metabolic Phenotype. Crit Rev Biochem Mol Biol (2018) 53:667–82. 10.1080/10409238.2018.1556578 30668176

[48] Sheng J Kohno S Okada N Okahashi N Teranishi K Matsuda F. Treatment of RB1-Intact Hepatocellular Carcinoma With CDK4/6 Inhibitor Combination Therapy. Hepatology (2021). 10.1002/hep.31872 33931882

[49] Li T Jiang S Han M Yang Z Lv J Deng C. Exogenous Melatonin as a Treatment for Secondary Sleep Disorders: A Systematic Review and Meta-Analysis. Front Neuroendocrinol (2019) 52:22–8. 10.1016/j.yfrne.2018.06.004 29908879

