# Supplementary material for: Development and Validation of Epigenetic Modification-Related Signals for the Diagnosis and Prognosis of Hepatocellular Carcinoma
Source: Front Oncol. 2021 Jun 17;11:649093. doi: 10.3389/fonc.2021.649093 (PMC8256693; doi:10.3389/fonc.2021.649093)
Supplement: Supplementary file 1 [file DataSheet_1.pdf]

1.Data processing

```
library("limma")
outTab=data.frame()
grade=c(rep(1,conNum),rep(2,treatNum))
rt=read.table(inputFile,sep="\t",header=T,check.names=F)
rt=as.matrix(rt)
rownames(rt)=rt[,1]
exp=rt[,2:ncol(rt)]
dimnames=list(rownames(exp),colnames(exp))
data=matrix(as.numeric(as.matrix(exp)),nrow=nrow(exp),dimnames=dimnames)
data=avereps(data)
for(i in row.names(data)){
   geneName=unlist(strsplit(i,"\\|",))[1]
   geneName=gsub("\\/", "_", geneName)
   rt=rbind(expression=data[i,],grade=grade)
   rt=as.matrix(t(rt))
   wilcoxTest<-wilcox.test(expression ~ grade, data=rt)
   conGeneMeans=mean(data[i,1:conNum])
   treatGeneMeans=mean(data[i,(conNum+1):ncol(data)])
   logFC=log2(treatGeneMeans)-log2(conGeneMeans)
   pvalue=wilcoxTest$p.value
   conMed=median(data[i,1:conNum])
   treatMed=median(data[i,(conNum+1):ncol(data)])
   diffMed=treatMed-conMed
      if( ((logFC>0) & (diffMed>0)) | ((logFC<0) & (diffMed<0)) ){

outTab=rbind(outTab,cbind(gene=i,conMean=conGeneMeans,treatMean=treatGeneMeans,logFC
=logFC,pValue=pvalue))
        }
}
pValue=outTab[,"pValue"]
fdr=p.adjust(as.numeric(as.vector(pValue)),method="fdr")
outTab=cbind(outTab,fdr=fdr)

write.table(outTab,file="all.txt",sep="\t",row.names=F,quote=F)

outDiff=outTab[(                    abs(as.numeric(as.vector(outTab$logFC)))>logFCfilter                    &
as.numeric(as.vector(outTab$fdr))<fdrFilter),]
write.table(outDiff,file="diff.xls",sep="\t",row.names=F,quote=F)

heatmap=rbind(ID=colnames(data[as.vector(outDiff[,1]),]),data[as.vector(outDiff[,1]),])
write.table(heatmap,file="diffGeneExp.txt",sep="\t",col.names=F,quote=F)
```

```
pdf(file="vol.pdf",height=5,width=5)
xMax=max(abs(as.numeric(as.vector(outTab$logFC))))
yMax=max(-log10(outTab$fdr))+1
plot(as.numeric(as.vector(outTab$logFC)), -log10(outTab$fdr), xlab="logFC",ylab="-log10(fdr)",
     main="Volcano", ylim=c(0,yMax),xlim=c(-xMax,xMax),yaxs="i",pch=20, cex=0.8)
diffSub=subset(outTab, fdr<fdrFilter & as.numeric(as.vector(logFC))>logFCfilter)
points(as.numeric(as.vector(diffSub$logFC)), -log10(diffSub$fdr), pch=20, col="red",cex=0.8)
diffSub=subset(outTab, fdr<fdrFilter & as.numeric(as.vector(logFC))<(-logFCfilter))
points(as.numeric(as.vector(diffSub$logFC)), -log10(diffSub$fdr), pch=20, col="green",cex=0.8)
abline(v=0,lty=2,lwd=3)
dev.off()
```

2. KEGG

```
library("clusterProfiler")
library("org.Hs.eg.db")
library("enrichplot")
library("ggplot2")

rt=read.table("id.txt",sep="\t",header=T,check.names=F)
rt=rt[is.na(rt[,"entrezID"])==F,]
gene=rt$entrezID

kk <- enrichKEGG(gene = gene, organism = "hsa", pvalueCutoff =0.05, qvalueCutoff =0.05)
write.table(kk,file="KEGGId.txt",sep="\t",quote=F,row.names = F)
```

3. GO

```
library("clusterProfiler")
library("org.Hs.eg.db")
library("enrichplot")
library("ggplot2")


rt=read.table("id.txt",sep="\t",header=T,check.names=F)
rt=rt[is.na(rt[,"entrezID"])==F,]                          因
gene=rt$entrezID

kk <- enrichGO(gene = gene,
               OrgDb = org.Hs.eg.db,
               pvalueCutoff =0.05,
               qvalueCutoff = 0.05,
               ont="all",
               readable =T)
write.table(kk,file="GO.txt",sep="\t",quote=F,row.names = F)
```

4. LASSO

```
library("glmnet")
library("survival")

rt=read.table("lassoInput.txt",header=T,sep="\t",row.names=1)

x=as.matrix(rt[,c(3:ncol(rt))])
y=data.matrix(Surv(rt$futime,rt$fustat))

fit <- glmnet(x, y, family = "cox", maxit = 1000)
pdf("lambda.pdf")
plot(fit, xvar = "lambda", label = TRUE)
dev.off()

cvfit <- cv.glmnet(x, y, family="cox", maxit = 1000)
pdf("cvfit.pdf")
plot(cvfit)
abline(v=log(c(cvfit$lambda.min,cvfit$lambda.1se)),lty="dashed")
dev.off()

coef <- coef(fit, s = cvfit$lambda.min)
index <- which(coef != 0)
actCoef <- coef[index]
lassoGene=row.names(coef)[index]
write.table(lassoGene,file="lassoGene.txt",sep="\t",quote=F,row.names=F,col.names=F)
```

5. WGCNA

```
library("WGCNA")                                   #引用 WGCNA 包
data=read.table("diffGeneExp.txt",sep="\t",header=T,check.names=F,row.names=1)
group=sapply(strsplit(colnames(data),"\\-"),"[",4)
group=sapply(strsplit(group,""),"[",1)
group=gsub("2","1",group)
data=data[,group==0]
datExpr0=t(data)
gsg = goodSamplesGenes(datExpr0, verbose = 3)
if (!gsg$allOK)
{
   # Optionally, print the gene and sample names that were removed:
   if (sum(!gsg$goodGenes)>0)
      printFlush(paste("Removing genes:", paste(names(datExpr0)[!gsg$goodGenes], collapse = ",
")))
   if (sum(!gsg$goodSamples)>0)
      printFlush(paste("Removing samples:", paste(rownames(datExpr0)[!gsg$goodSamples],
collapse = ", ")))
   # Remove the offending genes and samples from the data:
```

```
    datExpr0 = datExpr0[gsg$goodSamples, gsg$goodGenes]
}
sampleTree = hclust(dist(datExpr0), method = "average")
pdf(file = "1_sample_cluster.pdf", width = 12, height = 9)
par(cex = 0.6)
par(mar = c(0,4,2,0))
plot(sampleTree, main = "Sample clustering to detect outliers", sub="", xlab="", cex.lab = 1.5,
cex.axis = 1.5, cex.main = 2)
abline(h = 10000, col = "red")
dev.off()
clust = cutreeStatic(sampleTree, cutHeight = 10000, minSize = 10)
table(clust)
keepSamples = (clust==1)
datExpr0 = datExpr0[keepSamples, ]
traitData = read.table("mRNAsi.txt",row.names=1,header=T,comment.char = "",check.names=F)
fpkmSamples = rownames(datExpr0)
traitSamples =rownames(traitData)
sameSample=intersect(fpkmSamples,traitSamples)
datExpr0=datExpr0[sameSample,]
datTraits=traitData[sameSample,]

sampleTree2 = hclust(dist(datExpr0), method = "average")
traitColors = numbers2colors(datTraits, signed = FALSE)
pdf(file="2_sample_heatmap.pdf",width=12,height=12)
plotDendroAndColors(sampleTree2, traitColors,
                    groupLabels = names(datTraits),
                    main = "Sample dendrogram and trait heatmap")
dev.off()

enableWGCNAThreads()
powers = c(1:20)        sft = pickSoftThreshold(datExpr0, powerVector = powers, verbose = 5)
pdf(file="3_scale_independence.pdf",width=9,height=5)
par(mfrow = c(1,2))
cex1 = 0.9
plot(sft$fitIndices[,1], -sign(sft$fitIndices[,3])*sft$fitIndices[,2],
     xlab="Soft Threshold (power)",ylab="Scale Free Topology Model Fit,signed R^2",type="n",
     main = paste("Scale independence"));
text(sft$fitIndices[,1], -sign(sft$fitIndices[,3])*sft$fitIndices[,2],
     labels=powers,cex=cex1,col="red");
abline(h=0.90,col="red")
plot(sft$fitIndices[,1], sft$fitIndices[,5],
     xlab="Soft Threshold (power)",ylab="Mean Connectivity", type="n",
     main = paste("Mean connectivity"))
text(sft$fitIndices[,1], sft$fitIndices[,5], labels=powers, cex=cex1,col="red")
```

```
dev.off()
softPower =sft$powerEstimate
adjacency = adjacency(datExpr0, power = softPower)
TOM = TOMsimilarity(adjacency);
dissTOM = 1-TOM
geneTree = hclust(as.dist(dissTOM), method = "average");
pdf(file="4_gene_clustering.pdf",width=12,height=9)
plot(geneTree, xlab="", sub="", main = "Gene clustering on TOM-based dissimilarity",
     labels = FALSE, hang = 0.04)
dev.off()
minModuleSize = 50
dynamicMods = cutreeDynamic(dendro = geneTree, distM = dissTOM,
                            deepSplit = 2, pamRespectsDendro = FALSE,
                            minClusterSize = minModuleSize);
table(dynamicMods)
dynamicColors = labels2colors(dynamicMods)
table(dynamicColors)
pdf(file="5_Dynamic_Tree.pdf",width=8,height=6)
plotDendroAndColors(geneTree, dynamicColors, "Dynamic Tree Cut",
                    dendroLabels = FALSE, hang = 0.03,
                    addGuide = TRUE, guideHang = 0.05,
                    main = "Gene dendrogram and module colors")
dev.off()

MEList = moduleEigengenes(datExpr0, colors = dynamicColors)
MEs = MEList$eigengenes
MEDiss = 1-cor(MEs);
METree = hclust(as.dist(MEDiss), method = "average")
pdf(file="6_Clustering_module.pdf",width=7,height=6)
plot(METree, main = "Clustering of module eigengenes",
     xlab = "", sub = "")
MEDissThres = 0.25
abline(h=MEDissThres, col = "red")
dev.off()
merge = mergeCloseModules(datExpr0, dynamicColors, cutHeight = MEDissThres, verbose = 3)
mergedColors = merge$colors
mergedMEs = merge$newMEs
pdf(file="7_merged_dynamic.pdf", width = 9, height = 6)
plotDendroAndColors(geneTree, cbind(dynamicColors, mergedColors),
                    c("Dynamic Tree Cut", "Merged dynamic"),
                    dendroLabels = FALSE, hang = 0.03,
                    addGuide = TRUE, guideHang = 0.05)
dev.off()
moduleColors = mergedColors
```

```
table(moduleColors)
colorOrder = c("grey", standardColors(50))
moduleLabels = match(moduleColors, colorOrder)-1
MEs = mergedMEs

nGenes = ncol(datExpr0)
nSamples = nrow(datExpr0)
moduleTraitCor = cor(MEs, datTraits, use = "p")
moduleTraitPvalue = corPvalueStudent(moduleTraitCor, nSamples)
pdf(file="8_Module_trait.pdf",width=6,height=6)
textMatrix = paste(signif(moduleTraitCor, 2), "\n(",
                   signif(moduleTraitPvalue, 1), ")", sep = "")
dim(textMatrix) = dim(moduleTraitCor)
par(mar = c(5, 10, 3, 3))
labeledHeatmap(Matrix = moduleTraitCor,
               xLabels = names(datTraits),
               yLabels = names(MEs),
               ySymbols = names(MEs),
               colorLabels = FALSE,
               colors = blueWhiteRed(50),
               textMatrix = textMatrix,
               setStdMargins = FALSE,
               cex.text = 0.5,
               zlim = c(-1,1),
               main = paste("Module-trait relationships"))
dev.off()
modNames = substring(names(MEs), 3)
geneModuleMembership = as.data.frame(cor(datExpr0, MEs, use = "p"))
MMPvalue = as.data.frame(corPvalueStudent(as.matrix(geneModuleMembership), nSamples))
names(geneModuleMembership) = paste("MM", modNames, sep="")
names(MMPvalue) = paste("p.MM", modNames, sep="")
traitNames=names(datTraits)
geneTraitSignificance = as.data.frame(cor(datExpr0, datTraits, use = "p"))
GSPvalue = as.data.frame(corPvalueStudent(as.matrix(geneTraitSignificance), nSamples))
names(geneTraitSignificance) = paste("GS.", traitNames, sep="")
names(GSPvalue) = paste("p.GS.", traitNames, sep="")
for (trait in traitNames){
  traitColumn=match(trait,traitNames)
  for (module in modNames){
    column = match(module, modNames)
    moduleGenes = moduleColors==module
    if (nrow(geneModuleMembership[moduleGenes,]) > 1){
      outPdf=paste("9_", trait, "_", module,".pdf",sep="")
      pdf(file=outPdf,width=7,height=7)
```

```
      par(mfrow = c(1,1))
      verboseScatterplot(abs(geneModuleMembership[moduleGenes, column]),
                         abs(geneTraitSignificance[moduleGenes, traitColumn]),
                         xlab = paste("Module Membership in", module, "module"),
                         ylab = paste("Gene significance for ",trait),
                         main = paste("Module membership vs. gene significance\n"),
                         cex.main = 1.2, cex.lab = 1.2, cex.axis = 1.2, col = module)
      abline(v=0.8,h=0.5,col="red")
      dev.off()
    }
  }
}
probes = colnames(datExpr0)
geneInfo0 = data.frame(probes= probes,
                       moduleColor = moduleColors)
for (Tra in 1:ncol(geneTraitSignificance))
{
  oldNames = names(geneInfo0)
  geneInfo0 = data.frame(geneInfo0, geneTraitSignificance[,Tra],
                         GSPvalue[, Tra])
  names(geneInfo0) = c(oldNames,names(geneTraitSignificance)[Tra],
                       names(GSPvalue)[Tra])
}

for (mod in 1:ncol(geneModuleMembership))
{
  oldNames = names(geneInfo0)
  geneInfo0 = data.frame(geneInfo0, geneModuleMembership[,mod],
                         MMPvalue[, mod])
  names(geneInfo0) = c(oldNames,names(geneModuleMembership)[mod],
                       names(MMPvalue)[mod])
}
geneOrder =order(geneInfo0$moduleColor)
geneInfo = geneInfo0[geneOrder, ]
write.table(geneInfo, file = "GS_MM.xls",sep="\t",row.names=F)
```

六．Drug target

```
options("repos"= c(CRAN="https://mirrors.tuna.tsinghua.edu.cn/CRAN/"))
options(BioC_mirror="http://mirrors.tuna.tsinghua.edu.cn/bioconductor/")
install.packages("pRRophetic_0.5.tar.gz", repos = NULL, type = "source")
library(tidyverse)
library(ISOpureR)
library(impute)
library(pRRophetic)
```

```
library(SimDesign)
library(cowplot)
expr <- read.table("LIHC..txt",sep = "\t",row.names = 1,check.names = F,stringsAsFactors = F,header = T)
normsam <- colnames(expr[,which(substr(colnames(expr),11,12) == "11")])
tumosam <- colnames(expr[,which(substr(colnames(expr),11,12) == "01")])
maf <- read_tsv("data_mutations_mskcc.txt", comment = "#")
maf$Tumor_Sample_Barcode <- paste0("LIHC",substr(maf$Tumor_Sample_Barcode,8,15))
signature <- read.table("17gene.txt",sep = "\t",row.names = 1,check.names = F,stringsAsFactors = F,header = T)
dim(signature
auc <- read.table("CTRP_AUC_raw.txt",sep = "\t",row.names = NULL,check.names = F,stringsAsFactors = F,header = T) # Supplementary Data Set 3
auc$comb <- paste(auc$master_cpd_id,auc$master_ccl_id,sep = "-")
auc <- apply(auc[,"area_under_curve",drop = F], 2, function(x) tapply(x, INDEX=factor(auc$comb), FUN=max, na.rm=TRUE)) auc <- as.data.frame(auc)
auc$master_cpd_id <- sapply(strsplit(rownames(auc),"-",fixed = T),"[",1)
auc$master_ccl_id <- sapply(strsplit(rownames(auc),"-",fixed = T),"[",2)
auc <- reshape(auc,
               direction = "wide",
               timevar = "master_cpd_id",
               idvar = "master_ccl_id")
colnames(auc) <- gsub("area_under_curve.","",colnames(auc),fixed = T)
ctrp.ccl.anno <- read.table("CTRP_ccl_anno.txt",sep = "\t",row.names = NULL,check.names = F,stringsAsFactors = F,header = T) # Supplementary Data Set 1
ctrp.cpd.anno <- read.table("CTRP_cpd_anno.txt",sep = "\t",row.names = NULL,check.names = F,stringsAsFactors = F,header = T) # Supplementary Data Set 2
write.table(auc,"CTRP_AUC.txt",sep = "\t",row.names = F,col.names = T,quote = F)
ctrp.auc <- read.table("CTRP_AUC.txt",sep = "\t",row.names = 1,check.names = F,stringsAsFactors = F,header = T)
prism.auc <- read.delim("PRISM_AUC.txt",sep = "\t",row.names = 1,check.names = F,stringsAsFactors = F,header = T) sensitivity AUC (PRISM Repurposing Secondary Screen) 19Q4
prism.ccl.anno <- prism.auc[,1:5]prism.auc <- prism.auc[,-c(1:5)]
ctrp.auc <- ctrp.auc[,apply(ctrp.auc,2,function(x) sum(is.na(x))) < 0.2*nrow(ctrp.auc)]
prism.auc <- prism.auc[,apply(prism.auc,2,function(x) sum(is.na(x))) < 0.2*nrow(prism.auc)]
rmccl <- paste0("CCL",na.omit(ctrp.ccl.anno[which(ctrp.ccl.anno$ccle_primary_site == "haematopoietic_and_lymphoid_tissue"),"master_ccl_id"]))
rownames(ctrp.auc) <- paste0("CCL",rownames(ctrp.auc))
ctrp.auc <- ctrp.auc[setdiff(rownames(ctrp.auc),rmccl),]
ctrp.auc.knn <- impute.knn(as.matrix(ctrp.auc))$data
prism.auc.knn <- impute.knn(as.matrix(prism.auc))$data
ctrp.auc.knn <- ctrp.auc.knn/ceiling(max(ctrp.auc.knn)) prism.auc.knn <- prism.auc.knn/ceiling(max(prism.auc.knn))
```

```
ccl.expr <- read.table("CCLE_RNAseq_rsem_genes_tpm_20180929.txt",sep = "\t",row.names = 1,check.names = F,stringsAsFactors = F,header = T)
Ginfo <- read.table("overlapTable27.txt",sep = "\t",row.names = 1,check.names = F,stringsAsFactors = F,header = T)
ccl.expr <- ccl.expr[,-1]; rownames(ccl.expr) <- sapply(strsplit(rownames(ccl.expr),".",fixed = T),"[",1)
comgene <- intersect(rownames(ccl.expr),rownames(Ginfo))
ccl.expr <- ccl.expr[comgene,]
ccl.expr$gene <- Ginfo[comgene,"genename"]; ccl.expr <- ccl.expr[!duplicated(ccl.expr$gene),]; rownames(ccl.expr) <- ccl.expr$gene; ccl.expr <- ccl.expr[,-ncol(ccl.expr)]
```
