# Supplementary material for: Development and Validation of Epigenetic Modification-Related Signals for the Diagnosis and Prognosis of Hepatocellular Carcinoma
Source: Front Oncol. 2021 Jun 17;11:649093. doi: 10.3389/fonc.2021.649093 (PMC8256693; doi:10.3389/fonc.2021.649093)
Supplement: Supplementary file 2 [file DataSheet_2.pdf]

MATERIALS AND METHODS

Primers for PCR were used.

Gene Name	Forward Primer	Reverse Primer
β-actin (Human)	GTTGAGAACCGTGTACCATGT	TTCCCACAATTTGGCAAGAGC
RNASE4(Human)	TGCAGAGGACCCATTCATTGC	TCAAGTTGCAGTAGCGATCAC
GRHL2 (Human)	AGCGGAGCAAGTTCATTGGA	CTATCAAGGCCCAAAGGGGA

Application of antibody details

Antibody name	Company	Application
Gapdh (60004-1-Ig)	Proteintech	WB
Rnase4(ab200717)	Abcam	WB
GRHL2 (ab271023)	Abcam	WB/IHC
Rnase4(ab214293)	Abcam	IHC

Table S1. Five kinds of epigenetic regulatory factors

Transcription factor	M6A-related gene	Histone modification-related genes	RNA binding protein	DNA methylase
ADNP	METTL14	ASH1L	A1CF	DNMT3A
AFF4	METTL16	ATM	AAR2	DNMT1
AR	RBM15	ATR	AARS	DNMT3B
ARID3A	VIRMA	AURKB	AARS2	DNMT3L
ARNT	WTAP	BAZ1B	AARSD1	TET1
ARNTL	ZC3H13	BRCC3	ABCE1	TET2
ASCL1	FTO	BUB1	ABCF1	TET3
ASH2L	HNRNPA2B1	C14orf169	ABCF2	
ATF1	HNRNPC	CARM1	ABCF3	
ATF2	IGF2BP1	CDK17	ABL2	
ATF3	YTHDC1	CDK3	ABT1	
ATF4	YTHDC2	CDK5	AC004381.6	
ATF7	YTHDF1	CDY1B	ACIN1	
BACH1	YTHDF2	CDYL	ACLY	
BACH2	YTHDF3	CHUK	ACO1	
BATF	IGF2BP2	CLOCK	ACTBL2	
BCL11A	IGF2BP3	CREBBP	ACTN1	
BCL3		DAPK3	ADAD1	
BCL6		DOT1L	ADAD2	
BDP1		DTX3L	ADAR	
BHLHE40		DUSP1	ADARB1	
BMI1		EHMT1	ADARB2	
BRCA1		EHMT2	ADAT1	
BRD1		ELP3	ADAT2	
BRD2		EP300	ADAT3	
BRD3		EYA1	AEN	

BRD4	EYA2	AFF1
BRF1	EYA3	AFF2
BRF2	EZH1	AFF3
C17orf96	EZH2	AFF4
CBFB	GSG2	AGFG1
CBX2	GSK3B	AGO1
CBX3	GTF3C4	AGO2
CBX5	HAT1	AGO3
CBX7	HDAC1	AGO4
CBX8	HDAC10	AHNAK2
CDK2	HDAC11	AIMP1
CDK7	HDAC2	AIMP2
CDK8	HDAC3	AKAP1
CDK9	HDAC4	AKAP13
CDX2	HDAC5	AKAP17A
CEBPA	HDAC6	AKAP2
CEBPB	HDAC7	AKAP8
CENPA	HDAC8	AKAP8L
CHD1	HDAC9	AKNA
CHD2	HLCS	AKR1B1
CHD7	HUWE1	AL844220.1
CHD8	JAK2	ALAS2
CIITA	JHDM1D	ALKBH1
CPSF3L	JMJD5	ALKBH5
CREBBP	JMJD6	ALKBH8
CTNNB1	KAT2A	ALX4
CUX1	KAT2B	ALYREF
DNMT1	KAT5	ANG
DNMT3A	KDM1A	ANGEL1
DYRK1A	KDM1B	ANGEL2
E2F1	KDM2A	ANK3
E2F3	KDM2B	ANKHD1
E2F4	KDM3A	ANKRD17
E2F6	KDM3B	ANKRD27
E2F7	KDM4A	ANPEP
EBF1	KDM4B	ANXA1
EED	KDM4C	ANXA11
EGR1	KDM4D	ANXA7
EGR2	KDM5A	AP1G1
EHF	KDM5B	APBA3
EHMT2	KDM5C	APEX1
ELF1	KDM5D	API5
ELF5	KDM6A	APOBEC1
ELK1	KDM6B	APOBEC2

ELK4	LIMK2	APOBEC3B
ELL2	MAP3K8	APOBEC3F
EMX1	MASTL	APOBEC3G
EOMES	MGEA5	APOBEC3H
EP400	MLL	APOBEC4
EPAS1	MLL2	APTX
EPO	MLL3	AQR
ERCC6	MLL5	ARHGAP36
ERG	MYSM1	ARHGAP5
ESR1	MYST1	ARHGAP9
ESRRA	MYST2	ARHGEF28
ETS1	MYST3	ARL6IP4
ETV1	MYST4	ASCC1
EZH1	NAT10	ASCC3
EZH2	NCOA1	ASH1L
FLI1	NCOA3	ASNA1
FOS	NEK6	ASPH
FOSL1	NEK9	ATF7
FOSL2	NSD1	ATL3
FOXA1	PADI4	ATP13A1
FOXA2	PAK2	ATP6AP1
FOXK1	PARG	ATXN1
FOXM1	PARP1	ATXN10
FOXO1	PHF8	ATXN1L
FOXO3	PKN1	ATXN2
FOXP1	PPM1D	ATXN2L
FOXP2	PPP1CC	AUH
FOXP3	PPP2CA	AVL9
GABPA	PPP2CB	AZGP1
GATA2	PPP4C	BAG3
GATA3	PPP5C	BARD1
GATA4	PRDM2	BASP1
GATA6	PRDM9	BAZ2A
GATAD1	PRKCB	BAZ2B
GREB1	PRKCD	BCDIN3D
GRHL2	PRKDC	BCLAF1
GTF2B	PRMT1	BICC1
GTF2F1	PRMT2	BLVRB
GTF2I	PRMT5	BMS1
H2AFX	PRMT6	BOLL
HCFC1	PRMT7	BOP1
HDAC1	PRMT8	BRCA1
HDAC2	RAG1	BRIX1
HDAC3	RBX1	BSG

HDAC6
HEY1
HIF1A
HIRA
HNF1B
HNF4A
HNF4G
HOXA9
HOXB13
HOXB7
HOXC11
HOXC9
HSF1
HSF2
IKZF1
IRF1
IRF3
IRF4
IRF5
JARID2
JMJD1C
JMJD6
JUN
JUNB
JUND
KAT2B
KAT5
KDM1A
KDM2B
KDM3A
KDM4C
KDM5A
KDM5B
KDM5C
KDM6B
KLF11
KLF4
KLF5
LEF1
LHX2
LIN9
LMNA
LMNB1
LMO2

RING1
RNF168
RNF2
RNF20
RNF40
RNF8
RPS6KA4
RPS6KA5
SETD1A
SETD1B
SETD2
SETD7
SETD8
SETDB1
SETDB2
SETMAR
SIRT1
SIRT2
SIRT3
SIRT6
SMYD2
SMYD3
STK10
STK4
SUV39H1
SUV39H2
SUV420H1
SUV420H2
TLK1
UBR2
UHRF1
USP16
USP22
USP3
WHSC1
WHSC1L1
CDY1

BSPRY
BUD13
BYSL
BZW1
BZW2
C11orf57
C11orf58
C11orf67
C11orf68
C11orf80
C12orf43
C12orf65
C13orf26
C16orf42
C17orf37
C17orf85
C19orf29
C19orf43
C19orf53
C1D
C1orf144
C1orf55
C1QBP
C20orf27
C2orf15
C4orf29
C4orf43
C6orf170
C7orf30
C8orf33
C8orf59
C9orf114
C9orf129
C9orf23
C9orf6
C9orf78
C9orf80
CACTIN
CACYBP
CAD
CADM1
CALR
CALR3
CAND1

LYL1
MAF
MAFF
MAFK
MAX
MAZ
MBD2
MBD3
MECP2
MED12
MEF2A
MEF2B
MEF2C
MEIS1
MITF
MXI1
MYB
MYBL2
MYC
MYH11
NANOG
NCAPG
NCOR2
NFATC1
NFE2
NFIC
NFYA
NFYB
NIPBL
NOTCH1
NR1H2
NR2C2
NR2F1
NR2F2
NR3C1
NR4A1
NR5A2
NRF1
OGT
PAF1
PAX3
PAX5
PAX6
PBX1
CANX
CAPG
CAPRIN1
CAPRIN2
CARHSP1
CARS
CARS2
CASC3
CASK
CBFA2T2
CBX8
CC2D1A
CCAR1
CCAR2
CCDC114
CCDC129
CCDC43
CCDC59
CCDC86
CCDC87
CCDC88C
CCNC
CCNT1
CCNT2
CCRN4L
CCT3
CCT5
CCT8
CD200
CD2BP2
CD3EAP
CD55
CDC20
CDC37
CDC40
CDC42
CDC5L
CDHR3
CDK11A
CDK5RAP1
CDK9
CDKN2A
CDV3
CEBPZ

PBX3
PDX1
PHF8
PIAS1
PML
POLR2B
POLR3A
POLR3D
POLR3G
POU2F1
POU5F1
PPARD
PPARG
PRDM1
PRKDC
RAG1
RARA
RARG
RB1
RBBP5
RBL2
RBP2
RBPJ
RCOR1
RELA
RFX2
RFX5
RING1
RNF2
RUNX1
RUNX1T1
RXRA
RXRG
RYBP
SALL4
SAP30
SCML2
SETDB1
SF1
SFMBT1
SFPQ
SIN3A
SIRT6
SIX5

CELF1
CELF2
CELF3
CELF4
CELF5
CELF6
CEP170
CEPT1
CFL1
CGN
CHCHD7
CHD1
CHERP
CHORDC1
CHTF8
CHTOP
CIRBP
CLASP1
CLASP2
CLASRP
CLK1
CLK2
CLK3
CLK4
CLP1
CLTC
CMSS1
CMTR1
CMTR2
CNBP
CNN2
CNOT1
CNOT10
CNOT11
CNOT2
CNOT3
CNOT4
CNOT6
CNOT6L
CNOT7
CNOT8
CNP
CNTLN
COL5A2

SMAD1
SMAD2
SMAD3
SMAD4
SMARCA4
SMARCB1
SMARCC1
SMARCC2
SMC1A
SMC3
SNAI2
SNAPC2
SNAPC4
SOX17
SOX2
SOX9
SP2
SPDEF
SPIB
SRC
SREBF1
SREBF2
SRF
SSRP1
STAT1
STAT2
STAT3
STAT4
STAT5A
STAT5B
STAT6
SUMO1
SUMO2
SUPT5H
TAF1
TAL1
TAT
TBL1XR1
TBP
TCF12
TCF21
TCF7
TCF7L1
TCF7L2

COL7A1
COX4I1
COX5B
CPD
CPEB1
CPEB2
CPEB3
CPEB4
CPS1
CPSF1
CPSF2
CPSF3
CPSF3L
CPSF4
CPSF4L
CPSF6
CPSF7
CR925765.1
CRNKL1
CRYZ
CSDC2
CSDE1
CSTF1
CSTF2
CSTF2T
CSTF3
CTAG2
CTAGE5
CTIF
CTU1
CTU2
CWC15
CWC22
CWC25
CWC27
CWF19L1
CWF19L2
CXorf23
CYCS
DALRD3
DAP3
DARS
DARS2
DAZ1

TEAD1
TEAD4
TERF1
TERF2
TET2
TFAP2A
TFAP2C
THAP11
TP53
TP63
TP73
TRIM28
TTF2
UBTF
USF1
USF2
VDR
VEZF1
WDR5
WHSC1
WWTR1
XBP1
XRN2
YAP1
YY1
ZBTB17
ZBTB33

DAZ2
DAZ3
DAZ4
DAZAP1
DAZL
DBR1
DCAF13
DCP1A
DCP1B
DCP2
DCPS
DDX1
DDX10
DDX17
DDX18
DDX19A
DDX19B
DDX20
DDX21
DDX23
DDX24
DDX25
DDX26B
DDX27
DDX28
DDX31
DDX39A
DDX39B
DDX3X
DDX3Y
DDX4
DDX41
DDX42
DDX43
DDX46
DDX47
DDX49
DDX5
DDX50
DDX51
DDX52
DDX53
DDX54
DDX55

DDX56
DDX58
DDX59
DDX6
DDX60
DDX60L
DECR1
DEK
DENR
DGCR14
DGCR8
DHCR24
DHX15
DHX16
DHX29
DHX30
DHX32
DHX33
DHX34
DHX35
DHX36
DHX37
DHX38
DHX40
DHX57
DHX58
DHX8
DHX9
DICER1
DIDO1
DIMT1
DIP2B
DIS3
DIS3L
DIS3L2
DKC1
DLGAP1
DMXL1
DNAAF2
DNAH6
DNAJA3
DNAJC17
DNAJC21
DNAJC8

DNAJC9
DNAT1
DND1
DNM1L
DNMT1
DNMT3B
DNTTIP2
DPP10
DQX1
DRG1
DRG2
DROSHA
DSCAM
DSG1
DST
DTD1
DUS1L
DUS2
DUS2L
DUS3L
DUS4L
DUSP1
DUSP11
DUSP23
DXO
DYNC1H1
DYNC1I2
DYNLL1
DZIP1
DZIP1L
DZIP3
EARS2
EBNA1BP2
ECH1
ECT2L
EDC3
EDC4
EED
EEF1A1
EEF1A2
EEF1B2
EEF1D
EEF1E1
EEF1G

EEF2
EEF2K
EEFSEC
EFTUD1
EFTUD2
EIF1
EIF1AD
EIF1AX
EIF1AY
EIF1B
EIF2A
EIF2AK1
EIF2AK2
EIF2AK3
EIF2AK4
EIF2B1
EIF2B2
EIF2B3
EIF2B4
EIF2B5
EIF2C1
EIF2D
EIF2S1
EIF2S2
EIF2S3
EIF2S3L
EIF3A
EIF3B
EIF3C
EIF3CL
EIF3D
EIF3E
EIF3G
EIF3H
EIF3I
EIF3J
EIF3K
EIF3L
EIF3M
EIF4A1
EIF4A2
EIF4A3
EIF4B
EIF4E

EIF4E1B
EIF4E2
EIF4E3
EIF4ENIF1
EIF4G1
EIF4G2
EIF4G3
EIF4H
EIF5
EIF5A
EIF5A2
EIF5AL1
EIF5B
EIF6
ELAC1
ELAC2
ELAVL1
ELAVL2
ELAVL3
ELAVL4
ELOF1
EMG1
EML3
ENDOG
ENDOU
ENDOV
ENO3
ENOX1
ENOX2
EP300
EPB41
EPHB6
EPRS
EPS15L1
ERAL1
ERCC6
ERH
ERI1
ERI2
ERI3
ERMP1
ERN1
ERN2
ERO1LB

ERP29
ESF1
ESPL1
ESRP1
ESRP2
ETF1
ETFB
EVPLL
EWSR1
EXO1
EXOC1
EXOG
EXOSC1
EXOSC10
EXOSC2
EXOSC3
EXOSC4
EXOSC5
EXOSC6
EXOSC7
EXOSC8
EXOSC9
EYS
EZH2
FAM103A1
FAM120A
FAM120B
FAM120C
FAM184B
FAM192A
FAM195A
FAM38A
FAM46A
FAM50A
FAM55C
FAM98A
FAM98B
FAM98C
FARP1
FARS2
FARSA
FARSB
FASTK
FASTKD1

FASTKD2
FASTKD3
FASTKD5
FAU
FBL
FBLL1
FBXO17
FCF1
FDXACB1
FGF2
FIP1L1
FKBP10
FLNB
FLT1
FMNL1
FMR1
FNBP4
FRG1
FRG1B
FTH1
FTO
FTSJ1
FTSJ2
FTSJ3
FUBP1
FUBP3
FUS
FXR1
FXR2
FYTTD1
G3BP1
G3BP2
GADD45GIP1
GAPDH
GAR1
GARS
GATA4
GATC
GCFC2
GCLM
GDI2
GEMIN2
GEMIN4
GEMIN5

GEMIN6
GEMIN7
GEMIN8
GFM1
GFM2
GIGYF2
GKAP1
GLE1
GLG1
GLTSCR2
GMPPA
GMPR2
GNL1
GNL2
GNL3
GNL3L
GPATCH1
GPATCH4
GPATCH8
GPKOW
GPS1
GRSF1
GRWD1
GSDMB
GSDMD
GSPT1
GSPT2
GTF2F1
GTF2I
GTF3A
GTPBP1
GTPBP10
GTPBP2
GTPBP3
GTPBP4
GUF1
H1FX
HABP4
HADH
HARS
HARS2
HBS1L
HDLBP
HEATR1

HELZ
HELZ2
HENMT1
HES2
HEXIM1
HEXIM2
HINT3
HIP1
HIP1R
HMGN1
HN1L
HNRNPA0
HNRNPA1
HNRNPA1L2
HNRNPA2B1
HNRNPA3
HNRNPAB
HNRNPC
HNRNPCL1
HNRNPD
HNRNPDL
HNRNPF
HNRNPH1
HNRNPH2
HNRNPH3
HNRNPK
HNRNPL
HNRNPLL
HNRNPM
HNRNPR
HNRNPU
HNRNPUL1
HNRNPUL2
HP1BP3
HPDL
HPSE2
HRSP12
HSDL2
HSPA4
HSPB8
HSPH1
HTATSF1
IARS
IARS2

IBTK
ICT1
IDH1
IER5L
IFIH1
IFIT1
IFIT1B
IFIT2
IFIT3
IFIT5
IFRD1
IFT172
IGF2BP1
IGF2BP2
IGF2BP3
IGHMBP2
ILF2
ILF3
IMP3
IMP4
INADL
INTS1
INTS10
INTS12
INTS2
INTS3
INTS4
INTS5
INTS6
INTS7
INTS8
INTS9
INVS
IPO11
IPO13
IPO4
IPO5
IPO7
IPO8
IPO9
IQGAP1
IQSEC2
IREB2
IRF2BP2

ISG20
ISG20L2
ISY1
ITGB1
JAKMIP1
KARS
KAT8
KAZN
KBTBD3
KHDC1
KHDC1L
KHDRBS1
KHDRBS2
KHDRBS3
KHNYN
KHSRP
KIAA0020
KIAA0101
KIAA0391
KIAA0430
KIAA0664
KIAA0907
KIAA0922
KIAA1429
KIF11
KIF18A
KIF1B
KIF2A
KIF2C
KIN
KLHDC4
KPNA2
KPNA3
KPNB1
KRR1
KRT2
KYNU
L1CAM
L1TD1
LAMA2
LAMB1
LAMC1
LARP1
LARP1B

LARP4
LARP4B
LARP6
LARP7
LARS
LARS2
LAS1L
LCMT2
LENG9
LENG9
LIMCH1
LIN28A
LIN28B
LMNA
LONP1
LRPPRC
LRRC40
LRRC47
LRRFIP1
LRRFIP2
LSG1
LSM1
LSM10
LSM11
LSM12
LSM14A
LSM14B
LSM2
LSM3
LSM4
LSM5
LSM6
LSM7
LSMD1
LSP1
LTK
LUC7L
LUC7L2
LUC7L3
LUZP4
MACF1
MAEL
MAGED2
MAGOH

MAGOHB
MAK16
MAP1S
MAP7D3
MAPKAPK2
MAPRE1
MARS
MARS2
MAT1A
MATR3
MAZ
MBNL1
MBNL2
MBNL3
MCAM
MCAT
MCTS1
MDN1
MECP2
MEMO1
MEPCE
MESDC2
METAP1
METTL1
METTL10
METTL14
METTL2A
METTL2B
METTL3
METTL5
MEX3A
MEX3B
MEX3C
MEX3D
MGST3
MIF
MIF4GD
MKRN1
MKRN2
MKRN3
MLEC
MLLT3
MOCOS
MOGS

MOV10
MOV10L1
MPG
MPHOSPH10
MPHOSPH6
MRM1
MRP63
MRPL1
MRPL10
MRPL11
MRPL12
MRPL13
MRPL14
MRPL15
MRPL16
MRPL17
MRPL18
MRPL19
MRPL2
MRPL20
MRPL21
MRPL22
MRPL23
MRPL24
MRPL27
MRPL28
MRPL3
MRPL30
MRPL32
MRPL33
MRPL34
MRPL35
MRPL36
MRPL37
MRPL38
MRPL39
MRPL4
MRPL40
MRPL41
MRPL42
MRPL43
MRPL44
MRPL45
MRPL46

MRPL47
MRPL48
MRPL49
MRPL50
MRPL51
MRPL52
MRPL53
MRPL54
MRPL55
MRPL9
MRPS10
MRPS11
MRPS12
MRPS14
MRPS15
MRPS16
MRPS17
MRPS18A
MRPS18B
MRPS18B
MRPS18C
MRPS2
MRPS21
MRPS22
MRPS23
MRPS24
MRPS25
MRPS26
MRPS27
MRPS28
MRPS30
MRPS31
MRPS33
MRPS34
MRPS35
MRPS36
MRPS5
MRPS6
MRPS7
MRPS9
MRRF
MRTO4
MSI1
MSI2

MSL3
MSLNL
MT-ATP6
MT-CO2
MTERF
MTERFD1
MTERFD2
MTFMT
MTG1
MTHFSD
MTIF2
MTIF3
MTO1
MTPAP
MTRF1
MTRF1L
MVK
MVP
MXI1
MYEF2
MYH10
MYH11
MYO9A
MYST4
N4BP1
N6AMT1
NA
NA
NAA16
NAA38
NAF1
NANOS1
NANOS2
NANOS3
NAP1L1
NARS
NARS2
NAT10
NCBP1
NCBP2
NCBP2L
NCL
NCOA5
NCOR2

NDRG1
NDRG2
NEFH
NELFE
NFKB2
NFX1
NFXL1
NGDN
NHP2
NHP2L1
NID1
NIFK
NIP7
NKRF
NKX6-2
NLE1
NLRP12
NMD3
NMT1
NOA1
NOB1
NOC2L
NOC3L
NOC4L
NOL10
NOL11
NOL12
NOL3
NOL6
NOL7
NOL8
NOL9
NOLC1
NOM1
NONO
NOP10
NOP14
NOP16
NOP2
NOP56
NOP58
NOP9
NOVA1
NOVA2

NPM1
NPM2
NPM3
NR0B1
NSA2
NSRP1
NSUN2
NSUN3
NSUN4
NSUN5
NSUN6
NSUN7
NTPCR
NUCB1
NUDT16
NUDT16L1
NUDT21
NUFIP1
NUFIP2
NUMA1
NUP153
NUP155
NUP188
NUPL2
NUTF2
NXF1
NXF2
NXF2B
NXF3
NXF5
NXT1
NXT2
NYNRIN
OAS1
OAS2
OAS3
OASL
OBFC1
OBSL1
ODZ1
OPA1
OSTC
PA2G4
PABPC1

PABPC1L
PABPC1L2A
PABPC1L2B
PABPC3
PABPC4
PABPC4L
PABPC5
PABPN1
PABPN1L
PACSIN2
PAIP1
PAIP2
PAIP2B
PAK1IP1
PALLD
PAN2
PAN3
PAPD4
PAPD5
PAPD7
PAPOLA
PAPOLB
PAPOLG
PARK7
PARN
PARP1
PARP12
PARP4
PARS2
PARVA
PATL1
PATL2
PCBP1
PCBP2
PCBP3
PCBP4
PCDHAC1
PCDHB4
PCF11
PCMT1
PCNA
PCNT
PDCD11
PDCD4

PDCD5
PDCD6
PDCD6IP
PDCD7
PDE12
PDE4DIP
PDIA5
PDIA6
PEG10
PELO
PES1
PET112
PFDN6
PFN1
PGAM1
PGK1
PGRMC1
PHAX
PHB
PHF10
PHF3
PHF5A
PHRF1
PICALM
PIH1D1
PIH1D2
PIH1D3
PIN4
PINX1
PIP4K2C
PIWIL1
PIWIL2
PIWIL3
PIWIL4
PLD6
PLIN3
PLP2
PLRG1
PLS3
PNKP
PNLDC1
PNN
PNO1
PNPT1

PNRC2
POLDIP3
POLR1E
POLR2A
POLR2B
POLR2D
POLR2E
POLR2F
POLR2G
POLR2H
POLR2I
POLR2J
POLR2J2
POLR2J3
POLR2K
POLR2L
POLRMT
POP1
POP4
POP5
POP7
POR
PPA1
PPAN
PPARGC1A
PPARGC1B
PPFIA1
PPFIBP1
PPIE
PPIH
PPIL3
PPIL4
PPM1E
PPME1
PPP1CB
PPP1CC
PPP1R10
PPP1R10
PPP1R8
PPRC1
PPWD1
PQBP1
PRCC
PRDM2

PRDX1
PRDX2
PRDX3
PRDX6
PREB
PRIM1
PRKAR2A
PRKDC
PRKRA
PRMT1
PRMT5
PRPF18
PRPF19
PRPF3
PRPF3
PRPF31
PRPF38A
PRPF38B
PRPF39
PRPF4
PRPF40A
PRPF40B
PRPF4B
PRPF6
PRPF8
PRR3
PSIP1
PSMA1
PSMA6
PSMD2
PSMD9
PSPC1
PSTK
PTBP1
PTBP2
PTBP3
PTCD1
PTCD2
PTCD3
PTDSS1
PTGES3
PTGES3L-AARSD1
PTGS1
PTMS

PTPRF
PTPRM
PTRF
PTRH1
PTRH2
PTRHD1
PUF60
PUM1
PUM2
PURA
PURB
PURG
PUS1
PUS10
PUS3
PUS7
PUS7L
PUSL1
PWP1
PWP2
QARS
QKI
QRSL1
QSER1
QTRT1
QTRTD1
R3HCC1
R3HCC1L
R3HDM1
R3HDM2
RAB35
RABGAP1
RAD23B
RAE1
RALY
RALYL
RAN
RANBP10
RANBP17
RANBP2
RANBP6
RAP1GAP2
RAP1GDS1
RAPGEF6

RARS
RARS2
RASD1
RAVER1
RAVER2
RBBP4
RBBP6
RBBP7
RBFOX1
RBFOX2
RBFOX3
RBM10
RBM11
RBM12
RBM12B
RBM14
RBM15
RBM15B
RBM17
RBM18
RBM19
RBM20
RBM22
RBM23
RBM24
RBM25
RBM26
RBM27
RBM28
RBM3
RBM33
RBM34
RBM38
RBM39
RBM4
RBM41
RBM42
RBM43
RBM44
RBM45
RBM46
RBM47
RBM48
RBM4B

RBM5
RBM6
RBM7
RBM8A
RBMS1
RBMS2
RBMS3
RBMX
RBMX2
RBMXL1
RBMXL2
RBMXL3
RBMY1A1
RBMY1B
RBMY1D
RBMY1E
RBMY1F
RBMY1J
RBPMS
RBPMS2
RC3H1
RC3H2
RCC1
RCL1
RDM1
RECQL
REPIN1
REXO1
REXO2
REXO4
RIOK1
RIOK2
RIOK3
RNASE1
RNASE10
RNASE11
RNASE12
RNASE13
RNASE2
RNASE3
RNASE4
RNASE6
RNASE7
RNASE8

RNASE9
RNASEH1
RNASEH2A
RNASEH2B
RNASEH2C
RNASEK
RNASEL
RNASET2
RNF10
RNF113A
RNF113B
RNF17
RNF214
RNF32
RNGTT
RNH1
RNMT
RNMTL1
RNPC3
RNPS1
RP9
RPA1
RPF1
RPF2
RPGRIP1
RPL10
RPL10A
RPL10L
RPL11
RPL12
RPL13
RPL13A
RPL14
RPL15
RPL17
RPL18
RPL18A
RPL19
RPL21
RPL22
RPL22L1
RPL23
RPL23A
RPL24

RPL26
RPL26L1
RPL27
RPL27A
RPL28
RPL29
RPL3
RPL30
RPL31
RPL32
RPL34
RPL35
RPL35A
RPL36
RPL36A
RPL36AL
RPL37
RPL37A
RPL38
RPL39
RPL39L
RPL3L
RPL4
RPL41
RPL5
RPL6
RPL7
RPL7A
RPL7L1
RPL8
RPL9
RPLP0
RPLP1
RPLP2
RPP14
RPP21
RPP25
RPP25L
RPP30
RPP38
RPP40
RPRD2
RPS10
RPS11

RPS12
RPS13
RPS14
RPS15
RPS15A
RPS16
RPS17
RPS17L
RPS18
RPS19
RPS19BP1
RPS2
RPS20
RPS21
RPS23
RPS24
RPS25
RPS26
RPS27
RPS27A
RPS27L
RPS28
RPS29
RPS3
RPS3A
RPS4X
RPS4Y1
RPS4Y2
RPS5
RPS6
RPS7
RPS8
RPS9
RPSA
RPUSD1
RPUSD2
RPUSD3
RPUSD4
RQCD1
RRBP1
RRNAD1
RRP1
RRP12
RRP15

RRP1B
RRP36
RRP7A
RRP8
RRP9
RRS1
RSL1D1
RSL24D1
RSRC1
RSRC2
RTCA
RTCB
RTF1
RUVBL1
RUVBL2
RWDD4
S100A7
S100A8
S100P
SAFB
SAFB2
SAMD4A
SAMD4B
SAMHD1
SAP18
SARNP
SARS
SARS2
SART1
SART3
SBDS
SCAF1
SCAF11
SCAF4
SCAF8
SCAMP3
SDAD1
SDHA
SEC61A1
SECISBP2
SECISBP2L
SEPSECS
SERBP1
SERF2

SETD1A
SETD1B
SETD2
SETD7
SETX
SF1
SF3A1
SF3A2
SF3A3
SF3B1
SF3B14
SF3B2
SF3B3
SF3B4
SF3B5
SFPQ
SFSWAP
SHMT2
SHQ1
SIDT1
SIDT2
SKIV2L
SKIV2L
SKIV2L2
SLBP
SLC4A1AP
SLC4A2
SLC9A3R1
SLIRP
SLTM
SLU7
SMAD1
SMAD2
SMAD3
SMAD4
SMAD5
SMAD6
SMAD7
SMAD9
SMG1
SMG5
SMG6
SMG7
SMG8

SMG9
SMN1
SMN2
SMNDC1
SMOC1
SND1
SNIP1
SNRNP200
SNRNP25
SNRNP27
SNRNP35
SNRNP40
SNRNP48
SNRNP70
SNRPA
SNRPA1
SNRPB
SNRPB2
SNRPC
SNRPD1
SNRPD2
SNRPD3
SNRPE
SNRPF
SNRPG
SNRPN
SNUPN
SNW1
SON
SPAG9
SPARCL1
SPATS2
SPATS2L
SPCS2
SPEN
SPG20
SQSTM1
SRA1
SRBD1
SREK1
SREK1IP1
SRFBP1
SRI
SRP14

SRP19
SRP54
SRP68
SRP72
SRP9
SRPK1
SRPK2
SRPR
SRRM1
SRRM2
SRRM3
SRRM4
SRRT
SRSF1
SRSF10
SRSF11
SRSF12
SRSF2
SRSF3
SRSF4
SRSF5
SRSF6
SRSF7
SRSF8
SRSF9
SSB
SSR1
SSU72
STAT3
STAU1
STAU2
STRAP
STRBP
STT3B
SUB1
SUGP1
SUGP2
SUMO2
SUPT4H1
SUPT5H
SUPT6H
SUPV3L1
SURF2
SURF6

SUZ12
SWT1
SYCE1L
SYF2
SYMPK
SYNCRIP
SYNE2
TACO1
TAF15
TAF1L
TAF9
TARBP1
TARBP2
TARDBP
TARS
TARS2
TARSL2
TAX1BP1
TBL3
TBRG4
TCERG1
TCF25
TCOF1
TDP2
TDRD1
TDRD10
TDRD12
TDRD15
TDRD3
TDRD5
TDRD6
TDRD7
TDRD9
TDRKH
TEFM
TEP1
TERT
TEX10
TEX13A
TFAM
TFB1M
TFB2M
TFCP2
TFIP11

TGS1
THG1L
THOC1
THOC2
THOC3
THOC5
THOC6
THOC7
THRAP3
THUMPD1
THUMPD2
THUMPD3
TIA1
TIAL1
TIMM44
TIPARP
TKTL1
TLN1
TLR3
TLR7
TLR8
TMEM214
TMPO
TNKS1BP1
TNPO1
TNPO2
TNPO3
TNRC6A
TNRC6B
TNRC6C
TOE1
TOP1
TOP2A
TOP3B
TPI1
TPR
TRA2A
TRA2B
TRAF3IP2
TRDMT1
TRIM21
TRIM25
TRIM33
TRIM56

TRIM61
TRIM71
TRIP10
TRIT1
TRMT1
TRMT10A
TRMT10B
TRMT10C
TRMT11
TRMT112
TRMT12
TRMT13
TRMT1L
TRMT2A
TRMT2B
TRMT44
TRMT5
TRMT6
TRMT61A
TRMT61B
TRMU
TRNAU1AP
TRNT1
TROVE2
TRPT1
TRUB1
TRUB2
TSEN15
TSEN2
TSEN34
TSEN54
TSFM
TSN
TSNAX
TSR1
TSR2
TSR3
TST
TTC16
TTF2
TTK
TUBA1C
TUBB
TUBB2C

TUFM
TUT1
TWISTNB
TXLNG
TXNDC5
TXNL4A
TXNL4B
TYW1
TYW3
TYW5
U2AF1
U2AF1L4
U2AF2
U2SURP
UBA1
UBA52
UBAP2
UBAP2L
UBE2O
UBE2T
UBQLN1
UBTF
UHMK1
UNK
UNKL
UPF1
UPF2
UPF3A
UPF3B
URB1
URB2
URM1
USB1
USP10
USP16
USP19
USP39
USP42
UTP11L
UTP14A
UTP14C
UTP15
UTP18
UTP20

UTP23
UTP3
UTP6
VAMP4
VARS
VARS2
VARSL
VASH1
VIM
VKORC1
VPS13D
WAC
WARS
WARS2
WBP11
WBP4
WDFY3
WDR12
WDR19
WDR3
WDR33
WDR35
WDR36
WDR4
WDR43
WDR46
WDR5
WDR61
WDR70
WDR74
WDR83
WDTC1
WIBG
WIPF3
WRAP53
XAB2
XPNPEP3
XPO1
XPO4
XPO5
XPO6
XPO7
XPOT
XRCC1

XRCC6
XRN1
XRN2
YAP1
YARS
YARS2
YBX1
YBX2
YBX3
YIPF3
YRDC
YTHDC1
YTHDC2
YTHDF1
YTHDF2
YTHDF3
ZC3H10
ZC3H11A
ZC3H12A
ZC3H12B
ZC3H12C
ZC3H12D
ZC3H13
ZC3H14
ZC3H15
ZC3H18
ZC3H3
ZC3H4
ZC3H6
ZC3H7A
ZC3H7B
ZC3H8
ZC3HAV1
ZC3HAV1L
ZC3HC1
ZCCHC11
ZCCHC13
ZCCHC14
ZCCHC17
ZCCHC2
ZCCHC24
ZCCHC3
ZCCHC5
ZCCHC6

ZCCHC7
ZCCHC8
ZCCHC9
ZCRB1
ZDHHC1
ZFC3H1
ZFHX2
ZFP36
ZFP36L1
ZFP36L2
ZFP91
ZFPM2
ZFR
ZFR2
ZGPAT
ZMAT2
ZMAT3
ZMAT5
ZNF106
ZNF121
ZNF142
ZNF207
ZNF239
ZNF277
ZNF326
ZNF346
ZNF385A
ZNF473
ZNF498
ZNF579
ZNF593
ZNF598
ZNF622
ZNF638
ZNF668
ZNF692
ZNF706
ZNF768
ZNF831
ZNFX1
ZNHIT6
ZRANB2
ZRSR1
ZRSR2

FigureS1

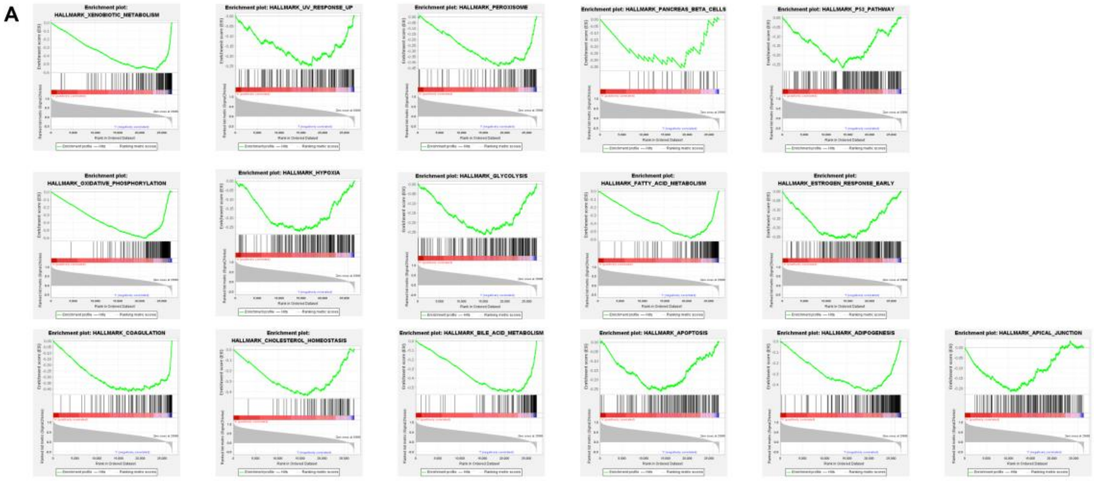

A. Intersection signal pathway of GSEA and GSVA analysis
